# Therapeutic Inhibition of Myc in Cancer. Structural Bases and Computer-Aided Drug Discovery Approaches

**DOI:** 10.3390/ijms20010120

**Published:** 2018-12-29

**Authors:** Lavinia A. Carabet, Paul S. Rennie, Artem Cherkasov

**Affiliations:** Vancouver Prostate Centre, University of British Columbia, 2660 Oak Street, Vancouver, BC V6H 3Z6, Canada; lcarabet@prostatecentre.com (L.A.C.); prennie@prostatecentre.com (P.S.R.)

**Keywords:** Myc-Max, cancer, therapeutic strategies, computer-aided drug discovery, small-molecule inhibitors, protein–protein interactions, protein-DNA interactions

## Abstract

Myc (avian myelocytomatosis viral oncogene homolog) represents one of the most sought after drug targets in cancer. Myc transcription factor is an essential regulator of cell growth, but in most cancers it is overexpressed and associated with treatment-resistance and lethal outcomes. Over 40 years of research and drug development efforts did not yield a clinically useful Myc inhibitor. Drugging the “undruggable” is problematic, as Myc inactivation may negatively impact its physiological functions. Moreover, Myc is a disordered protein that lacks effective binding pockets on its surface. It is well established that the Myc function is dependent on dimerization with its obligate partner, Max (Myc associated factor X), which together form a functional DNA-binding domain to activate genomic targets. Herein, we provide an overview of the knowledge accumulated to date on Myc regulation and function, its critical role in cancer, and summarize various strategies that are employed to tackle Myc-driven malignant transformation. We focus on important structure-function relationships of Myc with its interactome, elaborating structural determinants of Myc-Max dimer formation and DNA recognition exploited for therapeutic inhibition. Chronological development of small-molecule Myc-Max prototype inhibitors and corresponding binding sites are comprehensively reviewed and particular emphasis is placed on modern computational drug design methods. On the outlook, technological advancements may soon provide the so long-awaited Myc-Max clinical candidate.

## 1. Introduction

**Figure 1:**
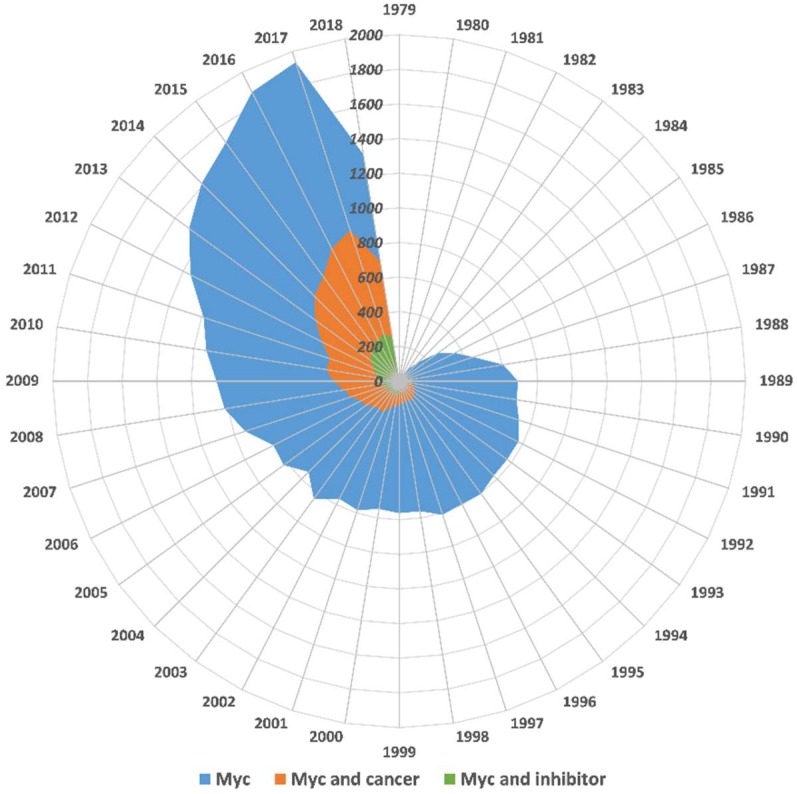
40 years of Myc research. Increase in the number of publications available in NCBI PubMed on Myc in general (blue), Myc in cancer (orange), and Myc inhibition (green) from 1979 to 2018.

Over 40 years have passed since the discovery of *MYC*, a major oncogene that is estimated to contribute to at least 75% of all human cancers, including prostate, breast, colon and cervical cancers, myeloid leukemia, lymphomas, small-cell lung carcinomas, and neuroblastoma, among others, most of which are aggressive and respond poorly to the current therapies [1] (Figure 1). Human c-Myc protein (hereafter Myc) is a nuclear transcription factor encoded by the *MYC* gene that is found at locus 8q24.21 in a broader region on chromosome 8, which is frequently amplified in cancers [2]. It was originally discovered as a homolog to a viral protein that causes avian leukemia [2,3]. Its two paralogs, N-Myc and L-Myc, which are encoded by *MYCN* and *MYCL* genes, were respectively identified in neuroblastoma and lung cancer as more tissue-specific factors [4,5,6].

Myc functions as a central downstream hub inside the nucleus, integrating signals from numerous upstream pathways to direct gene expression programs and regulate many biological functions, including promoting cell growth, proliferation, apoptosis, metabolism, and transformation while blocking differentiation [7,8,9,10,11,12]. Myc is under extremely tight control by the cell, but defects in its regulation lead to its overabundance and aberrant expression that are characteristic of many cancers [13]. Myc levels are controlled by multiple mechanisms, including negative autoregulation, gene expression, mRNA, and protein stability and degradation, which all become deregulated in human cancers [1,14,15].

Human Myc contains several highly conserved regions that are functionally important and are organized in the same fashion among the three Myc paralogs, including: a largely unstructured N-terminal transactivation domain (TAD) and an intrinsically disordered C-terminal region comprising the basic, helix-loop-helix, leucine zipper (bHLHLZ) dimerization, and DNA-binding domains [13]. These domains contain highly conserved modules that provide docking sites for a large number of cofactors that regulate Myc activity and stability [16,17]. Through its binding partners, Myc is able to regulate the chromatin landscape and elicit its oncogenic effects [18].

The obligate partner is Max, a homologous bHLHLZ Myc-associated factor [19,20]. Myc and Max are required to dimerize to undergo coupled folding and to adopt a stable helical conformation that forms the functional mandatory DNA-binding domain (DBD). Through the DBD, Myc and Max together bind specific DNA recognition sequences 5′-CACGTG-3′, termed E-boxes or enhancer-boxes, at enhancers and promoters of target genes. This triggers the recruitment of chromatin-remodeling complexes and assembly of the transcriptional machinery, thereby switching on more than 15% of the human genome on one hand and driving oncogenic transformation on the other [20]. The Myc-Max complex also binds DNA sites that vary from the palindromic hexanucleotide canonical sequence and are not bound with equal affinities. Non-canonical sequences, such as 5′-CACGCG-3′ and 5′-CATGGC-3′, represent low-affinity Myc-Max binding sites [21]. 

Myc is recognized as one of the most valuable targets in cancer, since an effective drug would make a very substantial impact in the field. However, for many years it has been pessimistically considered “undruggable” [22]. A major concern is that Myc inactivation by any drug may have undesirable side effects on normal cells [23]. Its nuclear localization represents another challenge. Moreover, Myc is an intrinsically disordered protein (IDP) that transiently acquires minimal secondary structure and exists as a “protein cloud” in a dynamic ensemble of unstable conformations, with no effective pockets on its surface [24]. The structural disorder of Myc (and Max, also an IDP) makes it difficult to characterize its interactions with ligands using experiments alone and importantly, is an inherent challenge in applying conventional structure-based drug design approaches to target its disordered structure [25]. A wide array of strategies has been employed to overcome these significant challenges in an attempt to reduce Myc-dependent oncogenic transformation. The diversity of Myc targeting approaches spans from targeting at all of its regulatory levels to targeting its protein–protein interactors, with some of these approaches yielding prototype inhibitors that have entered early phases of clinical trials [26,27,28,29,30,31]. However, the direct inhibition of Myc-Max interactions and binding to chromatin, aimed at blocking downstream gene expression characteristic of Myc-dependent tumors, has been unsuccessful to date despite almost three decades of research and development efforts [19]. 

In this review, we discuss Myc regulation and the structure-function relationships that are relevant for the development of inhibitors aimed at blocking Myc oncogenic transformation. We focus on structural aspects and the methodologies employed for binding site identification and discovery of structurally diverse direct small-molecule inhibitors of Myc-Max protein–protein and protein-DNA interactions, with special emphasis on recent computational approaches that hold great promise in accelerating drug discovery, lowering the costs, and enhancing translational potential into the clinic. 

## 2. Myc Regulation. Structure-Function Relationships

Myc is tightly regulated by the normal cells, but in many cancers this control is lost leading to its anomalous expression [1]. Myc deregulation occurs at any given stage across its short molecular life-cycle, from replication to transcription to its translation and degradation. Mechanisms that account for Myc deregulation include: amplifications or chromosomal translocations of the *MYC* locus that provoke its exacerbated expression, *MYC* mRNA destabilization through both direct and indirect regulatory events, and alteration in Myc protein turnover rate. The latter is due to either alterations in Myc protein stability normally dependent on Myc’s phosphorylation status but caused by mutations in key phosphorylation sites or alterations of expression of proteins that are involved in Myc’s post-translational modifications, such as altered signaling from important ubiquitin-ligase cofactors that engage the ubiquitin-proteasome system and lead to Myc protein degradation [1,13,32]. In addition, Myc’s aberrant expression occurs as a consequence of upstream oncogenic signals (e.g., Ras/MAPK, PI3K, Notch, Wnt), all converging on Myc [1,13,32]. Myc function is also highly dependent on chromatin context as well as binding partners and effector complexes that modulate various actions of Myc on gene expression [1,13,32].

### 2.1. Structure of MYC Gene and Protein. Regulatory Sites and Functional Domains

#### 2.1.1. *MYC* Gene Expression. Transcriptional and Posttranscriptional Control

**Figure 2:**
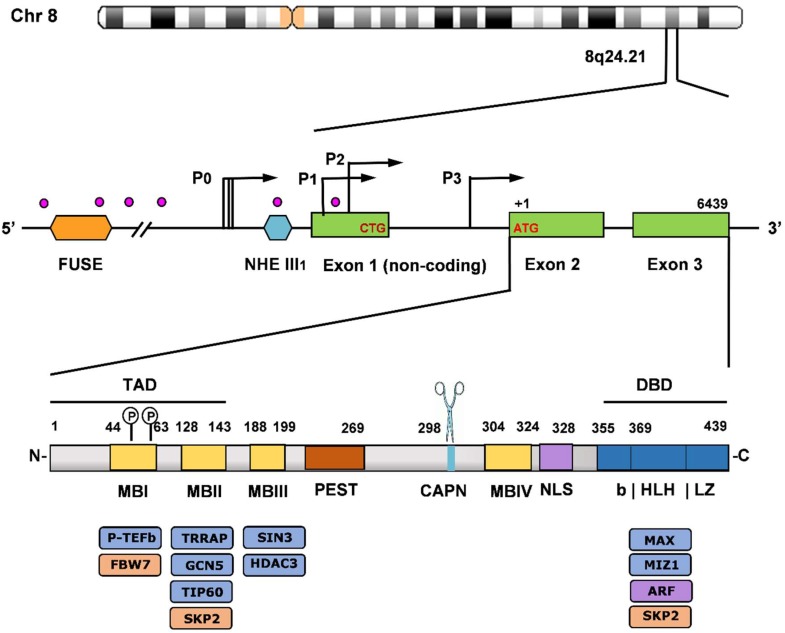
Structure of *MYC* gene and protein: functional domains and interactors. (**Top**) *MYC* locus. (**Middle**) *MYC* gene organization. (**Bottom**) Myc protein domains organization. A variety of proteins that regulate Myc activity and stability interact with these domains. The major Myc protein product, 439 amino acids long, is shown. Not drawn to scale. See text for details.

Human *MYC* gene, approximately 6 kbases long, found at locus 8q24.21 on chromosome 8, has an unusual topography. It contains three exons—a large non-coding exon 1, followed by coding exons 2 and 3, four distinct promoters—P_0_, P_1_, P_2_, and P_3_—that drive *MYC* transcription, two major translation start codons (CTG, and ATG), from which two universally expressed Myc proteins arise, two polyadenylation signals and several DNAse 1-hypersensitive sites (Figure 2) [32,33]. P_0_ transcripts start at multiple initiation sites. P_1_ and P_2_ are the two major classical TATA-containing promoter start sites located at the 5′ end of exon 1, with greater than three-quarters of *MYC* transcripts originating from the P_2_ promoter [33]. The *MYC* promoter region is a key convergence node spectacularly regulated by a myriad of signaling pathways, transcription factors, *cis*-regulatory elements, chromatin remodeling, and by its auto-suppression. The complexity of the *MYC* promoter control and the mechanisms accounting for it have been admirably and masterfully described in [33]. A few highlights follow. 

Nearly every major signal transduction pathway that controls cell proliferation or quiescence, impacts the *MYC* promoter, and regulates *MYC* transcription, either directly or indirectly. In turn, these pathways are activated by a broad range of signaling molecules, including mitogens, growth factors, hormones, cytokines, oncogenes, and tumor suppressors [33]. No single regulatory pathway accounts for the activation of the *MYC* promoter. On one hand, the pathways can cross-talk, are somewhat redundant and show variability dependent on cell type and cellular context. While being essential for normal Myc regulation in minimizing undesired Myc expression, the integrated biological output from the multiple-input signaling networks poses the risks of driving pathological conditions [33]. On the other hand, more than 30 transcription factors (such as E2F, SP1, β-catenin/TCF-4, Smad3, NF-κB, STAT3, ER, and AR) bind to the *MYC* promoter at distinct *cis*-regulatory sequences and act as integrative nodes at the *MYC* promoter and as effectors from the different signaling pathways, thus mediating the regulation of *MYC* transcription in response to various proliferative and anti-proliferative signals [33]. Two important *cis*-elements lie upstream of the P_1_ and P_2_ promoters—the far upstream sequence element (FUSE) and nuclease hypersensitivity element III 1 (NHEIII_1_)—that can form non-canonical DNA structures (such as repressive G-quadruplex configurations) and control *MYC* transcription in enthralling ways [33]. 

Moreover, *MYC* transcription is extensively regulated via chromatin remodeling and it depends on the presence or absence of particular nucleosomes, their histone acetylation, or methylation patterns, as well as the DNA methylation status [33]. Feedback loops to most, if not all, systems that are regulated by Myc, including Myc auto-suppression, provide important mechanisms for the control of Myc expression [33]. The Myc protein directly or indirectly affects its own expression level. The direct regulation involves a negative feedback loop where Myc protein represses its own major P_2_ promoter at the level of transcription initiation and concordantly the *MYC* promoter is occupied by the Myc protein itself. Myc auto-suppression requires Myc-Max heterodimerization, but it does not occur via binding of Myc-Max to the specific E-box, as the targeted promoter region lacks canonical 5′-CACGTG-3′ sequences, but instead occurs via binding to the Inr (initiator) element mediated by Inr-binding and E2F transcription factors [33]. Indirectly, Myc acts as both an activator and repressor of its own activators and repressors [33]. Additional factors that trigger the Myc-driven exacerbated cellular proliferation and transformation evidenced in cancer have been described in [32,33].

Posttranscriptional deregulatory events further contribute to transforming Myc [32]. Two mechanisms of *MYC* mRNA turnover have been reported. The first is translation-independent, involving poly(A) tail shortening that is regulated by AU-rich sequences in the 3′ untranslated region [34,35]. The second is a translation-dependent mechanism regulated by a region of mRNA corresponding to the C-terminal domain of the coding region determinant-binding protein (CRD-BP) [36]. Importantly, the stabilization of *MYC* mRNA by CRD-BP accounts for the increased mRNA stability observed in human cancers [37]. 

#### 2.1.2. Myc and Max Protein Organization and Interactors. Translational and Posttranslational Control

The major Myc protein product, a protein sequence of 439 amino acids, which migrates as p64 (i.e., at 64 kDa) starts with the ATG codon at the 5′ end of exon 2, while the minor protein product p67 having 14 additional N-terminal amino acids starts with the CTG initiation codon at the 3′ end of exon 1 [32,33]. Human Myc protein contains several highly conserved regions that are functionally important and organized in the same fashion among the three paralogs (c-Myc, N-Myc, and L-Myc) [13]. All Myc proteins possess a largely disordered N-terminal transactivation domain (TAD), a 143 amino acid domain that is required for transcriptional and cell-transforming activity and contains conserved functional modules, termed Myc boxes (MBI, MBII) (Figure 2 bottom). MBI serves as a phosphodegron and it is involved in the ubiquitination and proteasomal degradation of Myc [13]. Myc family members are very unstable with half-lives of 20–30 min in normal cells; however, in many tumors the stabilization of Myc contributes to its deregulation. Myc stability is controlled by multiple ubiquitin ligases. The E3 ubiquitin ligase FBW7 (F-Box and WD repeat domain containing 7) binds to MBI and regulates c-Myc and N-Myc in response to phosphorylation of Ser62 (stabilizes Myc) and Thr58 (destabilizes Myc) residues (these two major phosphorylation sites are indicated in Figure 2 above the MBI box). Mutation of Thr58 has been reported to occur in ~1/2 of Burkitt’s lymphoma cases, leading to Myc stabilization due to impaired proteasomal degradation and the evasion of apoptosis [38,39,40]. Loss of FBW7 has also been reported to result in decreased Myc turnover in a large number of tumors [41]. MBII, the most studied region within Myc TAD, is important for most Myc activities and it functions as a hub for binding to multiple key interactors, including TRRAP (transformation/transcription domain associated protein) involved in chromatin-dependent Myc transcriptional signaling [42]. TRRAP recruits chromatin-remodeling complexes, including histone acetyltransferases (HAT), such as GCN5 and Tip60, to promote gene activation. Acetylated, open chromatin is bound by bromodomain-containing proteins, such as BRD4, and other coactivators of the bromodomain and extra terminal (BET) family, which recruit the positive transcription elongation factor b (P-TEFb) complex at target genes promoters, activate the kinase activity of P-TEFb, which phosphorylates the C-terminal domain of RNA polymerase II (POLII), causing pause release and leading to transcriptional elongation [43]. Moreover, MBII is involved in Myc protein turnover as is a docking site for SKP2, the E3 ubiquitin ligase component S-phase kinase-associated protein 2 that, in addition to FBW7, is involved in the degradation of Myc [44,45]. SKP2 effects on Myc activity are briefly discussed below. The mechanisms underlying Myc degradation have been reviewed in detail by Farrell et al. [46]. 

Myc proteins also contain a central segment rich in proline, glutamic acid, serine, and threonine residues (PEST), which is necessary for rapid Myc degradation but not ubiquitination [39]. Two additional conserved Myc boxes are found in Myc proteins: MBIII is important for transcriptional repression, as it provides docking sites for components of histone deacetylase repressor complexes, such as SIN3 and HDAC3 [47,48], and MBIV, also important for Myc transcriptional activity and Myc-induced apoptosis. These boxes have been reported to interact with additional effectors, including CREB-binding p300/CBP transcriptional co-activators, and WDR5 (WD repeat-containing protein 5), which stabilizes Myc interactions with chromatin to promote target gene recognition and Myc-driven tumorigenesis [49]. Moreover, MBIV interacts with p27 (cyclin-dependent kinase inhibitor p27^KIP1^), one of the gatekeepers of G1-S transition of the cell cycle. p27 represses Myc and blocks Myc’s phosphorylation at Ser62, thus decreasing its activity. p27 is an essential target of SKP2 that triggers its ubiquitination and proteasomal-mediated degradation, hence relieving p27 repression of Myc and leading to transcriptional activation [50].

The calpain cleavage site (CAPN in Figure 2) is involved in cytosolic Myc partial cleavage of the C-terminus, resulting in “Myc-nick”, a 298 amino acid N-terminal segment that inactivates Myc transcriptional activity [51,52,53]. The nuclear localization sequence (NLS) is also implicated in Myc cellular-transforming activity, transcription, and apoptosis [54].

The C-terminal region of Myc proteins, ~100 amino acids in length, comprises the basic, helix-loop-helix, leucine zipper (bHLHLZ) dimerization, and DNA-binding (DBD) domains. The most important interactor at the C-terminal region is Max and as already mentioned, is mandatory for Myc transcriptional activation. An additional interactor with Myc’s C-terminus is Miz1 (Myc-interacting Zn-finger protein 1), with a role in Myc transcriptional repression. Myc repression involves the loss of Myc binding to E-box by the displacement of Max by other bHLHLZ proteins (see Section 2.2), allowing for Myc to associate with and sequester Miz1, a POZ domain-containing zinc finger protein that induces G1 cell cycle arrest [55,56]. Other important interactors with Myc C-terminus are ARF and SKP2. The ARF tumor suppressor antagonizes SKP2-mediated ubiquitination, inhibits Myc transactivation, proliferation, and transformation, and promotes Myc-induced, p53-independent apoptosis [57]. SKP2 recognizes Myc through both MBII and bHLHLZ motifs to promote Myc poly-ubiquitination and degradation. While SKP2 decreases Myc protein stability and stimulates its degradation, it has the opposite effect on Myc transcriptional activity, promoting it instead of inhibiting it as does FBW7 [46]. SKP2 is a direct target gene of Myc, which augments its expression. As such, SKP2 may contribute to oncogenesis by both enhancing Myc transcriptional activity and regulating its protein level [46]. Myc cofactors and their Myc-associated functions have been extensively covered in recent reviews [16,17]. 

### 2.2. The Extended Myc/Max/Mxd Network. The Players and Their Regulatory Functions

**Figure 3:**
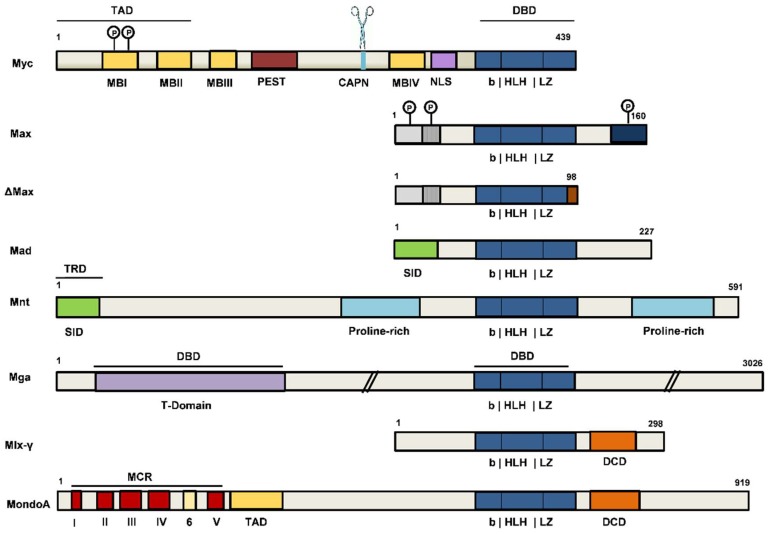
Structure-function schematic of the members of the extended Myc/Max network. Known functional domains of different network members are summarized. TAD: transactivation domain; DBD: DNA binding domain; P: position of known phosphorylation sites; SID: mSin3 interaction domain; TRD: trans-repression domain; T-domain: T-box DNA binding domain; DCD: dimerization and cytoplasmic localization domain. MCR: MondoA conserved region. Not drawn to scale. See text for details.

Myc and Max belong to an extended network of related bHLHLZ transcription factors that function as regulators of different aspects of cell behavior, as they mediate a broad transcriptional response to diverse signals, including mitogenic, growth arrest, and metabolic stimuli [13,55,58,59]. The transcriptional network is centered on two nodes, with the Max binding proteins forming one node, whereas Mlx binding proteins form a second node. Beside the Myc family members, the Max-centered network includes members of the Mxd (originally called Mad) family of bHLHLZ proteins, Mxd1-4, and the more distantly related Mnt (Figure 3). In addition, Max binds to Mga, the largest protein in the Max network and a “dual-specificity” transcription factor, in that possesses not only the bHLHLZ Max DNA binding motif, but also a T-domain DNA binding motif [56]. 

The Mxd family members act as antagonists of the Myc function. Mxd proteins, like Myc, do not homodimerize nor bind to DNA as monomers. Instead, Mxd and Mnt proteins form heterodimers with Max, but act as transcriptional repressors competing with Myc-Max heterodimer for binding at same promoter-proximal E-boxes. The transcriptional repression activity of Mxd and Mnt proteins stems from their ability to bind the large corepressor Sin3 histone deacetylase complex (Figure 3) [13,55,58,59]. All Mxd and Mnt proteins possess near their N-terminus a conserved amino acid region, termed the mSin3 interaction domain (SID), which directly interacts with one amphipathic α–helical (PAH) domain within Sin3 [60]. Sin3 interacts with class I histone deacetylases (HDAC1 and HDAC2), leading to transcriptional silencing [60]. While the Myc triad associates with the TRRAP-GCN5 coactivator complex, the Mxd family members recruit the Sin3-HDAC corepressor complex, suggesting their antagonistic behavior [13]. The opposed transcriptional activities of Myc and Mxd families have been attributed to three overlapping mechanisms: competition for available Max to form heterodimers, competition of heterodimers for E-box DNA binding sites, and activation or repression of bound target genes [13,55,58,59]. 

The Mga member of the extended network has been suggested to act as a tumor suppressor, with inactivating mutations detected in leukemia [61]. The Mnt factor antagonizes both Myc-stimulated proliferation and apoptosis, its pro-survival function being critical for Myc-driven tumorigenesis consistent with Mnt’s dominant physiological activity of opposing the pro-apoptotic activity elicited by Myc [10]. It has been demonstrated that the loss of Mnt induces similar effects as Myc overexpression, such as enhanced transcription of Myc target genes, resulting in accelerated proliferation, apoptosis, and transformation [62,63]. It has been proposed that Myc functions by the relief of Mnt repression [63,64]. 

Max can form unstable homodimers, and unlike Myc it lacks a transactivation domain (Figure 3). At physiological levels, Max homodimers fail to regulate transcription, but Max overexpression can lead to transcriptional repression [65,66]. Overexpressed Max has been shown to reduce Myc-induced carcinogenesis [67,68]. In human cancer, higher Max levels have been associated with better prognosis [69]. Max protein has several isoforms that are generated by alternative splicing. Besides the dominantly expressed Max isoform (i.e., 160 amino acids in length), ΔMax lacks the C-terminal 61 amino acids, which are replaced by five residues before ending with an alternative exon (Figure 3) [19,70]. Max phosphorylation (Figure 3) blocks Max homodimerization, but not heterodimerization with Myc. ΔMax is not phosphorylated and dimerizes with Myc augmenting its transforming activity [70].

The network extended further with the discovery of a Max-like bHLHLZ protein, Mlx, as a dimerization partner for a subset of Mxd family members, including Mxd1, Mxd4, and Mnt (Figure 3). Mxd-Mlx heterodimers interact with Sin3, bind E-box DNA sequences, and repress transcription similarly to Mxd-Max dimers [55,58,59]. While Mlx does not associate with Max or Myc family members, it dimerizes with two others partners, MondoA (MLXIP) and ChREBP (MondoB or MLXIPL), which are cytoplasmic-nuclear shuttling proteins whose accumulation in the nucleus is triggered by glucose-derived metabolites [59,71]. MondoA-Mlx and ChREBP-Mlx heterodimers bind E-boxes, act as nutrient-sensing transcription factors, and regulate genes that are involved in glucose and glutamine metabolism, both fundamental biological processes in both normal and cancer cells [71].

The Myc triad does not dimerize with any member of the network other than Max, while only Max and Mlx can form homodimers. Moreover, only Myc proteins and MondoA carry a TAD to transactivate target genes. In terms of dimerization with Max, Myc, and Mxd network members bind Max with different efficiencies and distinct subcellular localization patterns between Myc-Max and Mxd-Max have been reported [72]. Recent studies revealed that Myc and Mnt compete for binding to limited amounts of Max, whose availability is subsequently modulated by the turnover of Mxd proteins, typically displaying short half-lives, very much like Myc, due to ubiquitin-mediated proteasomal degradation [13,55,58,59]. In contrast, MondoA and B are stable proteins and the tight regulation of transcriptional activity occurs through their nuclear accumulation in response to changes in metabolic flux [13,55,58,59,71]. The observed changes in the abundance of individual network players have functional consequences that are attributed to the competition for available Max and Mlx core members as well as for DNA-binding sites, and may account in part for the extraordinary tight regulation of Myc expression [13,55,58,59].

### 2.3. Structural Aspects of Protein–Protein and Protein-DNA Interactions within the Myc/Max/Mad Network and Beyond

**Figure 4:**
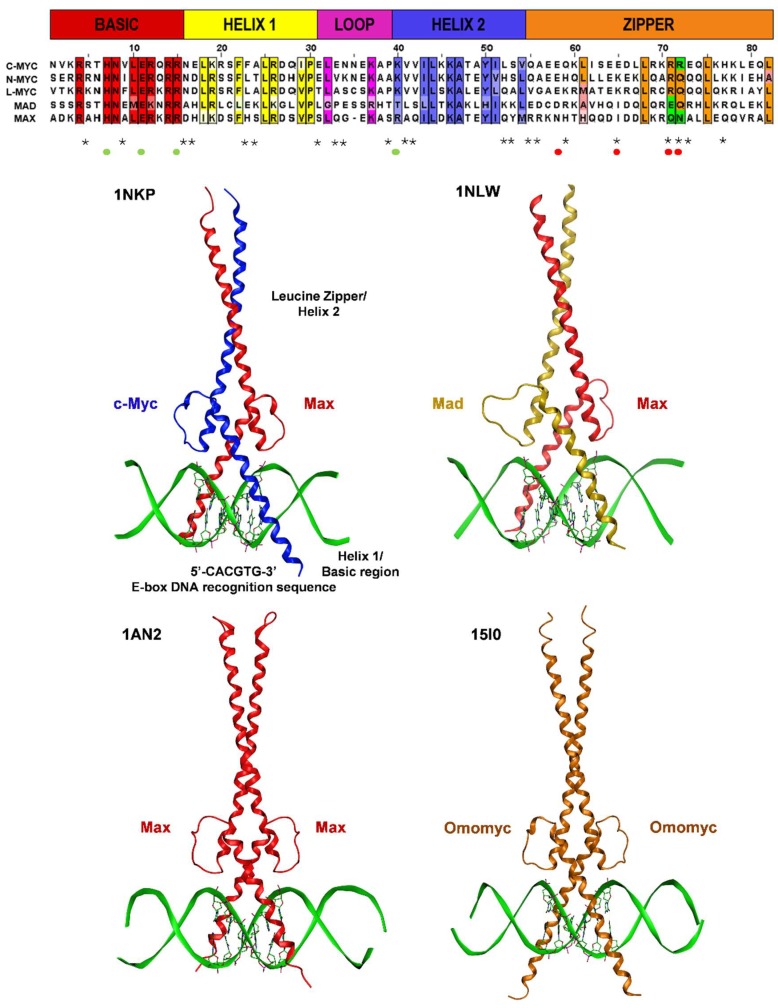
(**Top**) Multiple sequence alignment of the basic, helix-loop-helix, leucine zipper (bHLHLZ) domains of selected members of the Myc/Max/Mad network. Myc and Max monomers share ~60% similarity at the protein level in their bHLHLZ domains. Sequence conservations among Myc, Max, and Mad proteins are boxed and highlighted using the same coloring scheme as in the schematic of the bHLHLZ domain at the top. Conservation anomalies between the bHLHLZ domains of Max and Mad family relative to the three Myc paralogs [84] are indicated with a star (*) below the multiple alignment. Green dots indicate critical DNA recognition residues while red dots indicate important dimerization residues mutated in Omomyc. (**Bottom**) Available X-ray structures of complexes bound to the canonical E-box DNA recognition sequence: Myc-Max (PDB ID: 1NKP, 1.8 Å resolution), Mad-Max (PDB ID: 1NLW, 2 Å), Max-Max (PDB ID: 1AN2, 2.9 Å), and Omomyc (PDB ID: 15I0, 2.7 Å). Dimer assembly occurs through the helix-loop-helix (HLH) and leucine zipper (LZ) regions, while DNA binding takes place mainly through the basic (b) region and extends into the HLH region.

Available crystallographic data provided significant insights into the structural determinants of molecular interactions that govern the preferential assembly of homo- and heterodimers within the extended Myc network, as well as specific DNA recognition events [73]. The X-ray structure of bHLHLZ domain of the Max homodimer bound to the 5′-CACGTG-3′ E-box DNA sequence (PDB ID: 1AN2) was the first to reveal the overall ordered topology of the bHLHLZ domain (Figure 4), and it established the structural bases for DNA recognition [74]. Ten years later, the X-ray structures of Myc-Max (PDB ID: 1NKP) and Mad-Max heterodimers (PDB ID: 1NLW) were resolved, re-enforcing the secondary structure composition of the Max homodimer [75]. As shown in Figure 4, the Myc/Max/Mad bHLHLZ domains consist of two contiguous α-helices that are separated by a random loop. The first α-helical ordered element comprises residues from the basic (b) region and helix 1 (H1). The conserved proline residue at position 30 in the multiple alignment (shown at the top of Figure 4) terminates H1 and drives the loop formation connecting the two structured α-helical segments, the latter being composed of the helix 2 (H2) and the leucine zipper (LZ) regions [73,74,75]. The Max homodimer and the Myc- and Mad-Max heterodimers consist of two bHLHLZ monomers that fold into a parallel, four-helix bundle. The two basic regions, protruding from the N-termini of the bundle, insert into a modified B-form DNA conformation, characterized by a narrowed major groove and a widened minor groove, to make sequence specific contacts with the cognate E-box [73,74,75]. The two helical segments at the C-termini form the parallel, coiled-coil, or LZ dimerization domain. A well-defined globular core is formed by the conserved hydrophobic residues residing in the H1 and H2 helices of the four-helix bundle known to stabilize the Max-Max homodimer, distinguishing the bHLHLZ domain from that of purely coiled-coil LZ proteins [76]. Substitutions of Myc helix-loop-helix (HLH) and LZ motifs with the corresponding regions of structurally related proteins that do not interact with Max demonstrated that all of the conserved hydrophobic amino acids within H1 and H2 (Figure 4), in addition to the LZ motif, are required for specific Myc-Max dimer formation [77]. 

Extensive hydrophobic and polar interactions between the HLH and LZ regions stabilize both Max-Max homodimers as well as quasi-symmetric Myc-Max and Mad-Max heterodimer structures that noticeably differ in the corresponding coiled-coil LZ regions. The Max-Max complex contains a packing defect that is introduced by the charge-neutral Gln91-Asn92/Gln91-Asn92 pairing tetrad occurring at the C-termini of both Max monomers (Max monomer numbering) that promotes Max-Max homodimerization, in contrast to the positively charged pairing Arg423-Arg424/Arg423-Arg424 tetrad that disfavors the formation of the Myc-Myc homodimer. In the quasi-symmetric coiled-coil structures of Myc-Max and Mad-Max, the defect is compensated by charge complementarity at equivalent residues, with H-bonding interactions between Gln91-Asn92 pair with the positively charged Arg423-Arg424 pair (Myc monomer numbering) in Myc-Max heterodimer, and with the Glu125-Gln126 pair in the Mad-Max heterodimer, respectively, resulting in the tighter packing of the heterodimers [73,74,75]. These residues are highlighted in green and are boxed in the multiple sequence alignment at the top of Figure 4. 

The Max-Max, Myc-Max, and Mad-Max X-ray structures (Figure 4) further reveal three main segments that are responsible for specific DNA recognition at residue-level: residues from the basic and loop regions and the first residue of H2. Notably, three invariant residues within the basic region make base-specific contacts with the DNA 5′-CACGTG-3′ recognition sequence: the His, Glu, and Arg residues at positions 28, 32, and 35 in Max monomer-based numbering, at positions 359, 363, and 367 in Myc monomer-based numbering, and at positions 61, 65, and 69 in Mad (equivalent residues following the numbering in the Myc-Max dimer structure are His207, Glu211, and Arg215 in Max, and His906, Glu910, and Arg914 in Myc). These critical residues are highlighted in Figure 4 with green dots below the multiple sequence alignment. One H-bond between the conserved His and the central guanine of the E-box dictates the specificity for a purine base at that position. The invariant Glu makes two H-bonds with the adenine and the cytosine at positions 2 and 3 in the E-box sequence. Substitutions of the Glu to Gln, Asp, or Leu have been shown to abolish DNA binding [78]. The location of the Glu in the DNA major groove made DNA recognition by the shorter acidic side chain of aspartate incompatible [78]. The conserved Arg H-bonds the central guanine and in addition interacts with the phosphate group between the cytosine and the adenine at the first and second positions in the hexanucleotide dictating the identity of the central 5′-CG-3′ dinucleotide and the specificity for bHLHLZ proteins that bind the canonical class B E-box from those that have a hydrophobic amino acid at that position that bind non-canonical class A site, 5′-CAGCTG-3′ [78]. It has been shown that the substitution of this conserved Arg for Met suffices to convert some class A proteins (e.g., AP4, homologous bHLHLZ transcription factor) into a canonical class B E-box binding protein [79]. The basic region also makes a large number of contacts with phosphate groups spanning the entire backbone of the E-box. In the Myc-Max structure, several additional contacts have been described between residues that are specific to Myc and the phosphate backbone of the DNA, including Lys902 and Arg903. In addition, non-specific DNA contacts are contributed from Lys918 in the H1 region, Lys936 in loop region. Lastly, specific contacts are contributed from Lys939, the first residue in H2. Substitutions of its equivalent Max residue, Arg239, for Glu or Ala abolished both dimerization and DNA binding. Arg239 makes both side-chain and backbone amide contacts with DNA phosphate groups, and as such, tethers H2 to DNA and stabilizes the interactions between H1 and H2 by packing against the conserved Phe222 (Phe921 in Myc) located in H1 [74,75]. Only the Myc-Max heterodimer but not the Mad-Max counterpart was shown to form a bivalent hetero-tetramer capable of upregulating gene expression at distant promoters bearing widely separated E-boxes [75].

Further structural evidence on the specificity of DNA recognition as well as dimerization determinants came from the recently resolved X-ray structure of the Omomyc homodimer [80]. The comparison between the protein-DNA interfaces of Omomyc of Jung et al. [80] to that of the Myc-Max DNA complex of Nair et al. [75] demonstrated that both complexes bind to the DNA major groove with alike-formed scissor structures at the E-box, and that the basic region of both complexes assume the same phosphate-backbone and base-specific contacts with DNA. These contacts recapitulated the three invariant residues His, Glu, and Arg at positions 12, 16, and 20 in Omomyc as critical DNA recognition points. Omomyc is a dominant negative 93 residue bHLHLZ Myc protein fragment specifically designed to introduce four single-point mutations in the dimerization domain that correspond to four charged amino acids shown to prevent Myc homodimerization due to major steric and electrostatic clashes [81]. The Arg423-Arg424 pair (discussed above) that disfavors Myc homodimerization due to charge repulsion is replaced in Omomyc with the residues found in the Max sequence at these positions that favor Max homodimerization, instead: a glutamine and an asparagine, respectively. In addition, two glutamic acid residues at positions 410 and 417 in Myc are replaced in Omomyc by a threonine and isoleucine, respectively (with the latter also found in Max at the same position). A mutant with all four amino acids substituted with the Max-specific residues was found to homodimerize only weakly due to an unfavorable shape complementarity of amino acids around position 410 [81]. These mutations are highlighted in Figure 4 with red dots bellow the multiple sequence alignment.

Omomyc preferentially forms homodimers that are additionally stabilized by multiple interactions that are not present in the Myc-Max heterodimer, and introduced by specifically designed mutations [80]. Differences in protein–protein interactions between Omomyc and Myc-Max dimers extend beyond the mutated residues to the entire interface, making the Omomyc homodimer more stable than Myc-Max by an estimated free energy difference of −9 kcal/mol [80]. Omomyc can form dimers with both Myc and Max, but, due to repulsive interactions or lack of stabilizing interactions, they are less stable [80]. Omomyc homodimers effectively compete with Myc-Max heterodimers for binding to DNA on low-affinity promoters that are invaded by oncogenic Myc levels, while they do not outcompete binding of Myc-Max to promoters that are highly occupied at physiological levels of Myc [80]. In the latter case, binding of Myc-Max to chromatin appears to be stabilized by protein–protein interactions involving the Myc transactivation domain [80]. Omomyc effectively sequesters Myc away from the DNA and occupies the E-box with transcriptionally inactive dimers (i.e., Omomyc/Omomyc and Omomyc/Max). 

It is important to outline that Omomyc can effectively interfere with oncogenic Myc function by inhibiting gene expression that is characteristic of Myc-dependent tumors [80]. It has been demonstrated that Omomyc selectively targets the Myc protein interactions in that it binds c- and N-Myc, Max, and Miz-1, but it does not bind Mad or others HLH proteins [82]. Omomyc specifically prevents Myc binding to promoter E-boxes and transactivation of target genes while retaining Miz1-dependent binding to promoters and transregression, mechanisms of action accompanied by broad epigenetic changes [82]. Thus, in the presence of Omomyc, the Myc interactome is directed toward repression and its activity is switched from an oncogenic to a tumor suppressive one [82]. Noteworthy, and as a preamble to the Myc-Max inhibition described in the next section, Omomyc served as proof-of-concept that inhibiting Myc-Max interactions and its transcriptional output is an effective therapeutic strategy for cancer treatment. Omomyc established the feasibility of intermittent systemic inhibition of Myc-Max protein–protein and protein-DNA interactions for it showed efficacy against tumors with no toxicity to normal tissues, conferring indefinite survival in animal models [23,83]. As such, Omomyc alleviated the major concern about the undesired, deleterious side effects that Myc inhibition might have on healthy proliferating tissues.

## 3. Myc Targeting Approaches

A vast array of strategies, both direct and indirect, have been employed for targeting Myc by exploiting its multiple regulatory mechanisms, including *MYC* transcription and mRNA stability, Myc protein stability and degradation, as well as Myc binding to its interactome. Some of these approaches have yielded prototype inhibitors that have entered early clinical trials [29]. Examples include inhibitors of *MYC* transcription with direct G-quadruplex stabilizers, antisense oligonucleotides that induce *MYC* mRNA degradation, aberrant splicing of *MYC* pre-mRNA or translation block, as well as short-interfering RNAs [29,30].

Indirect Myc suppression has also been achieved via inhibitors of regulators of Myc protein stability and turnover (e.g., GSK3, Ras/Raf/MAPK, PP2A, FBW7, SKP2, hTERT) [29,30], inhibitors of pathways that are involved in Myc translation (e.g., MAPK, mTORC1 and FOXO3a) [30], and inhibitors of Myc chromatin remodeling and transcription of BET bromodomain proteins [85]. In the latter category, JQ1 was the first reported compound to inhibit Myc-associated chromatin remodeling enzyme Brd4 [86], followed by novel BET inhibitors, such as ZEN-3694, which entered clinical trials and demonstrated efficacy in a variety of solid tumors and hematological malignancies, alone or in combination with several standards [87], and more recent OTX015 [88] and TEN-010 [89]. Indirect suppression of *MYC* transcription and destabilization of the Myc protein in human Burkitt’s lymphoma has been recently achieved by targeting the Myc-HSP90 axis with HSP90 inhibitors (i.e., 17-AAG or 17-DMAG) [90]. Moreover, indirect synthetically lethal approaches have been reported. The synthetically lethal compound dihydroartemisinin (a common metabolite of the highly potent and safe anti-malarial agent, artemisinin) was found to activate the Ser/Thr kinase GSK3β, which in turn phosphorylates and destabilizes Myc [91].

Since c-Myc and N-Myc are highly-similar both structurally and functionally, their targeting approaches are equally similar, with perhaps an additional angle of indirect targeting N-Myc protein stability and turnover by antagonizing Aurora kinase A (AURKA) with small-molecule inhibitors that block AURKA/N-Myc interactions and promote N-Myc degradation. MLN8237 (Alisertib) and CD532 kinase inhibitors induce an allosteric transition in AURKA that results in conformational changes that destabilize N-Myc and triggers its phosphorylation at the N-terminus, its ubiquitylation, and ultimately its proteasomal degradation through the FBW7 ubiquitin ligase [92,93,94]. For in-depth coverage of strategies targeting both c-Myc and N-Myc, their preclinical stage, and clinical applicability, the reader is directed to several recent publications [29,30,95].

### 3.1. Small-Molecule Myc-Max Inhibitors

Although significant efforts have been made in the past 20 years, no approved small molecule drugs have been developed that directly block Myc-Max interactions or binding of the complex to DNA. A number of structurally-diverse small molecule prototypes have been reported but they lack in vivo efficacy and/or demonstrate suboptimal safety profiles [27,29]. On one hand, these shortcomings may arise from the lack of rational drug design efforts, as typically such chemicals emerge from high-throughput screening of limited chemical libraries. On the other hand, the disordered nature of the Myc protein and the lack of high-quality structures of ligated Myc-Max complexes further restrain therapeutic development efforts.

To summarize this section, it is possible to divide known Myc-Max small-molecule inhibitors into two categories (will be described in details in the following subsections):

1. those that act by interfering with protein–protein interactions and block heterodimerization of Myc with Max; and,
2. those that directly block Myc-Max binding to DNA.

Importantly, both types of inhibitors reduce the abundance of the Myc protein and inhibit the proliferation of several human cancer cell lines where they provoke an energy crisis that is marked by ATP depletion, neutral lipid accumulation, AMPK (adenosine monophosphate-activated protein kinase) activation, cell-cycle arrest, and apoptosis [96].

#### 3.1.1. Direct Myc-Max Protein–Protein Interactions Inhibitors

The first reported inhibitor of Myc-Max protein–protein interactions, IIA6B17, was identified by Berg et al. (2002) [97] from a combinatorial library of approximately 7000 peptidomimetic compounds that have been screened in vitro using the fluorescence resonance energy transfer (FRET) technique. Out of four initially identified inhibitors of Myc-Max heterodimerization, compound IIA6B17 was the most effective in suppressing the growth of Myc-transformed chicken embryo fibroblasts (CEF). Unfortunately, IIA6B17 also inhibited the transformation induced by Jun, a related basic zipper (bZip) transcription factor, suggesting a lack of specificity. Furthermore, Berg et al. [97] did not indicate whether the compound induced apoptosis. These observations limited the prospective of IIA6B17 as a drug candidate. Further, Shi et al. [98] introduced structural modification to the members of the same peptidomimetic library and identified two optimized compounds, Mycmycin-1 and Mycmycin-2, which were shown to specifically inhibit Myc-induced oncogenic transformation having no undesired effect on Jun-driven or unrelated Src protein kinase-driven transformation.

**Figure 5:**
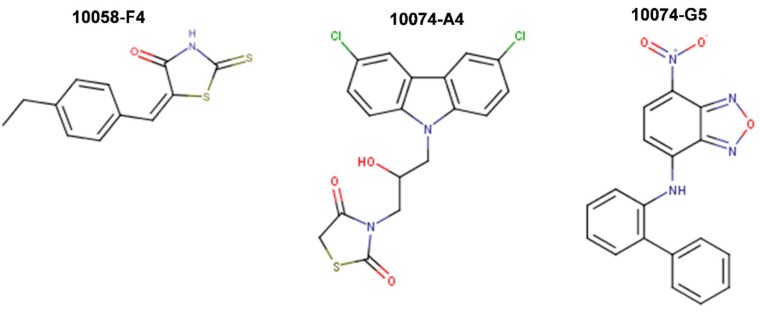
Chemical structures of 10058-F4, 10074-A4, and 10074-G5 inhibitors of Myc-Max dimerization identified through high-throughput screening of a finite combinatorial library. See text for details.

In 2003, Yin et al. [99] utilized the yeast two-hybrid system to screen 10,000 drug-like compounds from the Chembridge DIVERSet combinatorial library and identified seven low-molecular-weight inhibitors that blocked the Myc-Max interaction at the HLHLZ interface and demonstrated no significant cellular toxicity. Notably, three compounds—10058-F4, 10074-G5, and 10074-A4 (Figure 5)—demonstrated complete specificity toward Myc-Max. In particular, these compounds specifically inhibited Myc transcriptional activity and decreased cell growth of Myc-transformed rat fibroblasts. While the effect of the chemicals on cell viability was similar between Myc-parental lines expressing endogenous levels of Myc (i.e., TGR-1 cells having intact Myc alleles, Myc^+/+^) and Myc-overexpressing cells, HO15.19 Myc-null cell line (i.e., rat fibroblasts with homozygous deletion of the endogenous Myc gene, Myc^−/−^) showed a relative lack of response. Treatment with the Myc-specific compounds led to G0/G1 cell cycle arrest, followed by apoptosis.

Thus, compounds 10058-F4, 10074-A4, and 10074-G5 served as Myc prototypical inhibitors for many subsequent years, and helped accelerating research on therapeutic targeting of Myc. Nonetheless, while effective in cell lines, the use of these chemicals in vivo has been limited by their low potency (IC_50_s of 41.1 μM and 22.5 μM for 10058-F4 and 10074-G5, respectively, on growth inhibition of HL-60 human promyelocytic leukemia cells that overexpress Myc due to gene amplification) [100], lack of selectivity, and poor pharmacokinetic behavior as they undergo rapid metabolism resulting in low tumoral concentrations that are insufficient to block Myc-Max interactions in vivo, thus restricting their clinical applicability [101,102]. For instance, 10058-F4 characterization in vivo in severely combined immunodeficient (SCID) mice bearing DU145 and PC3 human prostate cancer xenografts, demonstrated the lack of efficacy and poor pharmacokinetic behavior of the specific Myc inhibitor in these classical models of moderate to high metastatic potential prostate cancer. Upon single intravenous dose treatment of mice with 20 or 30 mg/kg of 10058-F4, peak tumor concentrations of 10058-F4 were at least 10-fold lower than peak plasma concentrations, eight metabolites were identified in plasma, liver, and kidney, and no significant inhibition of tumor growth has been observed [101]. Similarly, 20 mg/kg of 10074-G5 administered as single intravenous dose to mice bearing Daudi Burkitt’s lymphoma xenografts showed that the compound reached a peak plasma concentration of 58 μM with a plasma half-life of ~37 min [102]. As in the case of 10058-F4, the peak tumor concentrations were at least 10-fold lower than peak plasma values and as many as 19 inactive metabolites were observed with a number of these appearing to be glucuronide derivatives of hydroxylated or nitro-reduced 10074-G5, products of primary and secondary metabolic biotransformation in the liver [102,103]. Therefore, while well-tolerated, the in vivo efficacy of 10058-F4 and 10074-G5 against the tested tumors was hampered by rapid metabolism and insufficient tumoral concentrations to block Myc-Max dimerization in vivo.

In a subsequent work, Wang et al. (2007) [104] attempted to develop more potent and selective analogs of 10058-F4 by employing computer-assisted chemical similarity search among >500,000 drug-like molecules from the Chembridge database as well as by direct chemical synthesis. The approach taken was to identify similar compounds that maintained >85% substructure similarity, but bore modifications in either the six-member or the five-member rhodanine ring of 10058-F4. 63 compounds were prioritized for IC_50_ determination in an MTT cell proliferation assay using HL-60 Myc-overexpressing cell line for comparison with that of 10058-F4. Four out of 48 identified six-member-substituted analogs had IC_50_s that were comparable to that of 10058-F4, ranging from 23 to 51 μM and four out of the 15 five-member ring analogs had improved potency (IC_50_ range 4.6-18 μM). These analogs were also able to disrupt Myc-Max heterodimerization in HL-60 cells as well as DNA recruitment, as measured by co-immunoprecipitation (co-IP) and electrophoretic mobility shift (EMSA) assays. 17 more analogs were generated by combining the best five- and six-member substitutions, but they did not result in further improvements. This inconsistent behavior possibly reflected multiple binding modes of singly or dual substituted analogs to different conformations of the intrinsically disordered Myc monomer [104].

**Figure 6:**
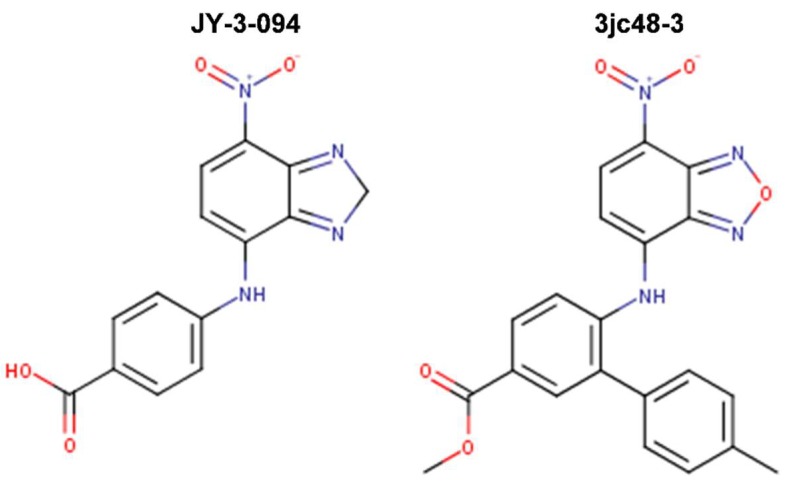
Chemical structures of JY-3-094 and 3jc48-3, improved analogs of compound 10074-G5.

Attempted optimizations of 10074-G5 in the form of JY-3-094 derivative (Figure 6) and its ester prodrug forms have also resulted in limited success. Thus, structure-activity relationship (SAR) studies of 10074-G5 led to the generation of an analog, JY-3-094, showing a stronger ability to disrupt the association between recombinant Myc and Max proteins [100,105], but it did not solve the issue of poor cell penetration. The nuclear magnetic resonance (NMR) model of 10074-G5 in its Myc binding site, discussed further below, was used as a guide. JY-3-094 retained the electron-rich nitro group and the furazan ring of 10074-G5, which were found to be critical for Myc inhibition [105], and included a para-carboxylic acid as a replacement for the ortho-phenyl ring of the ortho-biphenyl of 10074-G5, modification that led to enhanced activity relative to 10074-G5. In EMSA, JY-3-094 disrupted Myc-Max dimerization with an IC_50_ of 33 μM, which is significantly lower than IC_50_ = 146 μM established for 10074-G5. However, due to its ionizable carboxylic moiety, JY-3-094 failed in exhibiting any cytotoxicity to HL-60 and Daudi cells. This lack of activity was remedied by esterification of the para-carboxylic acid of JY-3-094 that resulted in a panel of ester prodrugs that enhanced cellular uptake (IC_50_ in low micromolar range in both HL-60 and Daudi cells), but unfortunately it impaired the ability to disrupt Myc-Max association in vitro [100]. Replacement of the carboxylic acid of JY-3-094 for a phenol ester resulted in compound SF-4-017, which showed comparable potency with the parent in vitro but showed inhibitory activity of Myc-Max dimerization in cells in co-IP, potentially being resistant to esterification [100]. Also related to 10074-G5 is the small molecule 3jc48-3 (Figure 6), a congener that showed increased potency and stability in cell-based assays [106]. 3jc48-3 was ~5 times as potent (IC_50_ = 34 μM) at inhibiting Myc-Max dimerization as the parent compound. 3jc48-3 exhibited an approximate two-fold selectivity for Myc-Max heterodimer over the Max-Max homodimer, suggesting binding to Myc. 3jc48-3 inhibited the proliferation of Myc-overexpressing HL-60 and Daudi cells with single-digit micromolar IC_50_ values by causing growth arrest at the G0/G1 phase. Co-IP studies indicated that 3jc48-3 inhibits Myc-Max dimerization in cells, further substantiated by specific Myc-driven gene silencing. Finally, 3jc48-3′s intracellular half-life was >17 h [106]. 3jc48-3 is considered to be one of the most potent, cellular active, and stable Myc inhibitors from the classical series developed by the group [100,103,105,106].

The most recent efforts to increase potency and selectivity as well as the translational potential of classical Myc inhibitors focus on medicinal chemistry and novel delivery technologies. Based on reversible linkage of chemically modified 10058-F4 and 10074-G5 to produce larger molecules, well-suited for targeting the Myc-Max dimer surface and capitalize on the drug-like properties of the small molecule components, Wanner et al. (2015) [107] generated intracellular self-assembly dimeric inhibitors that showed improved potency and activity in cancer cell lines overexpressing Myc (such as Daudi and Raji Burkitt’s lymphoma human cell lines). Moreover, Soodgupta et al. (2015) [108] reported nanoparticle targeted-delivery of 10058-F4 as an Sn2 lipase-labile pro-drug in the form of MI1-PD, which showed in vivo efficacy, as it extended survival in a mouse model of metastatic multiple myeloma.

**Figure 7:**
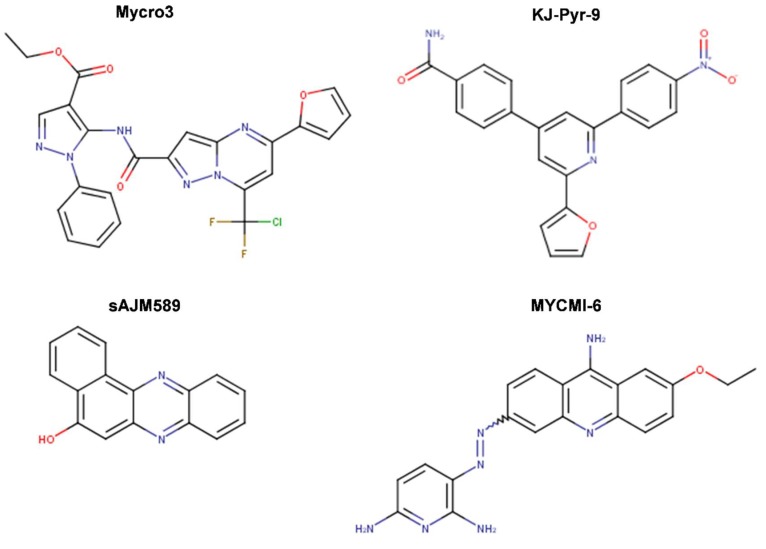
Chemical structures of Mycro3, KJ-Pyr-9, sAJM589, and MYCMI-6 inhibitors of Myc-Max dimerization identified through high-throughput screening of diverse small-sized compound libraries.

Further screens have identified small molecule inhibitors of protein–protein interactions of Myc-Max, such as Mycro3 and KJ-Pyr-9, which had improved pharmacokinetics, bioavailability and overall in vivo activity and demonstrated efficacy in mouse models of pancreatic and breast cancers. Mycro3 (Figure 7) was identified by Kiessling et al. (2007) [109] via high-throughput screening of a library of 1438 pyrazolo [1,5-α] pyrimidines that were built upon the two predecessor compounds, Mycro1 and Mycro2 [110]. Mycro3 was the first inhibitor from the constructed library that interfered with the formation of the Myc-Max-DNA complex by inhibiting protein–protein interactions between Myc and Max, which potently and preferentially interacted with Myc-Max over the Max-Max dimer or the related bZip AP-1 transcription factors (Jun/Fos proteins), and also without affecting the DNA binding of structurally unrelated STAT3 transcription factor to its binding site. Importantly, Mycro3 strongly inhibited Myc-dependent proliferation of U-20S osteosarcoma cells (70% reduction), while not inhibiting the Myc-Max-independent PC-12 cell line that lacks Max.

A subsequent study [111] demonstrated that the strength of Mycro3 activity was cell-line dependent, with TGR-1 cells having intact Myc alleles (Myc^+/+^) showing higher sensitivity to Mycro3 (IC_50_ of 0.25 μM) in comparison with other cell lines, such as HO15.19 Myc-null cells (Myc^−/−^) of common origin to TGR-1 (IC_50_ of 9 μM) or U-20S cells (IC_50_ of 10 μM). Moreover, in contrast to 10058-F4, which lacks antitumor activity in vivo, the pharmacokinetic profile of Mycro3 was quite good and characterized by sustained presence in circulation in mice at concentrations adequate for efficacy studies (0.5 μM). Although water-insoluble, in mice Mycro3 was amenable to daily administration by oral gavage as an emulsion for treatment of oncogenic *KRAS* (KRAS*)-induced pancreatic ductal adenocarcinoma (PDA), which is dependent on Myc activity. Stellas et al. (2014) [111] showed that genetic ablation of *MYC* effectively prevented the development of KRAS*-induced pancreatic, mammary and prostatic adenocarcinoma and penile squamous cell carcinoma in mice (consistent with Omomyc data). Treatment with Mycro3 Myc-inhibitor resulted in marked shrinkage of PDA and increased survival in a PDA mouse model, increased apoptosis, and reduced cell proliferation. Tumor growth was also significantly attenuated in Mycro3-treated mice carrying orthotopic or heterotopic xenografts of human pancreatic cells [111].

KJ-Pyr-9, a more recent inhibitor, was found by Hart et al. (2014) [112] within a 220-membered Kröhnke pyridine combinatorial library [113] screened by a fluorescence polarization assay for the inhibition of Myc-Max dimerization (Figure 7). By employing backscattering interferometry given the low aqueous solubility of the compounds, Hart et al. [112] determined that KJ-Pyr-9 binds directly to disordered Myc with nanomolar affinity (K_D_ of 6.5 nM) as well as to the Myc–Max heterodimer dissociating it (13.4 nM), but only weakly to the Max homodimer (>1 μM). Despite its low solubility, KJ-Pyr-9 was cell-permeable and it interfered with the Myc-Max complex formation in cells, as determined by a Renilla luciferase-based protein fragment complementation assay (PCA) (with Myc_332__–__439_–Max biosensor), supporting the direct intracellular binding of the compound to Myc.

It has been demonstrated that KJ-Pyr-9 preferentially blocks the proliferation of Myc-overexpressing cells (i.e., P493-6 engineered human B-cell line that overexpresses Myc in absence of tetracycline (Tet-off), leading to robust cell proliferation), with no or weak effects on the oncogenic activity of unrelated Src and Jun proteins. The compound also inhibited the proliferation of other cell lines that are dependent on increased Myc activity: NCI-H460 (large-cell lung cancer), MDA-MB-231 (breast adenocarcinoma), and SUM-159PT (estrogen-independent breast cancer) with IC_50_ values in the 5 to 10 μM range. The proliferation of Burkitt’s lymphoma cell lines with constitutively high expression of Myc, was more sensitive to KJ-Pyr-9 (IC_50_ between 1 and 2.5 μM). In addition, the proliferation of leukemia cell lines K-562, MOLT-4, and HL-60 overexpressing Myc was strongly inhibited, while the colon carcinoma cell line SW-480 was not affected. Importantly, KJ-Pyr-9 was also effective in blocking N-Myc-dependent proliferation. Moreover, KJ-Pyr-9 induced apoptosis by the cleavage of caspase 3, and specifically reduced well-established Myc-driven transcriptional signature in the P493-6 Myc-off cell line [114,115,116]. The compound showed promising pharmacokinetic properties in mouse (three mice injected at 10 mg/kg intraperitoneal) and in rat (1 mg/kg intravenous) models achieving blood concentrations sufficient to prompt its further investigation in vivo (mice: 3.5 μM in plasma, and 12.4 μM in brain, rat: elimination half-life in plasma ~1.84 h). It was not acutely toxic at doses as high as 10 mg/kg in mice, it crossed the blood-barrier, and it was present at higher concentration in brain tissue than in the blood after four hours. In vivo, KJ-Pyr-9 effectively blocked the growth of xenografts of MDA-MB-231 breast cancer cells in mice treated daily by intraperitoneal injection with 10 mg/kg of the compound. KJ-Pyr-9 represents one promising chemical probe for investigating the modulation of Myc-Max protein–protein interactions as an anticancer strategy. Combinations of KJ-Pyr-9 with other targeted drugs have been suggested for potentially enhanced effect on cancer cells [112]. Despite the results reported by Hart et al. [112], the binding mode of KJ-Pyr-9 to Myc remains to be determined to help explain the selectivity of the compound for Myc and additional studies in multiple animal models are required to address some limitations of their in vivo observations, including an examination of normal rapidly proliferating tissues in a manner similar to Omomyc treatment in healthy tissues [112].

sAJM589 is a novel small molecule Myc inhibitor identified by Choi et al. (2017) [117] from a Gaussia-based PCA-based high-throughput screen of ~400,000 drug-like molecules (Figure 7). sAJM589 is a potent disruptor of Myc-Max dimerization, a protein–protein interactions (PPI) inhibitor, with an IC_50_ of 1.8 μM, ~25-fold more potent than 10058-F4 in the PCA biochemical assay. Similar to KJ-Pyr-9, sAJM589 preferentially inhibited cellular proliferation of P493-6 (Tet-Off) cell line in a dose-dependent manner with IC_50_ = 1.9 μM, while in the presence of tetracycline, its IC_50_ was >20 μM. sAJM589 also showed a dose-dependent inhibition of various Myc-dependent cancer cell lines: Ramos (Burkitt’s lymphoma), HL-60 and KG1a (acute myeloid leukemia) with IC_50_s of 0.9, 1.2, and 0.8 μM, respectively, but had no effect on resting macrophages whose proliferation is independent of Myc activity. Co-IP confirmed the disruption of Myc-Max PPI and no effect on the Jun/Fos dimerization. In EMSA, over 2 μM of sAJM589 completely disrupted Myc-Max binding to E-box DNA. Moreover, biolayer interferometry (BLI) showed direct binding of sAJM589 to the LZ region (amino acids 403-437) of a biotin-tagged Myc protein in a dose dependent manner (6.25 to 100 μM sAJM589 concentration range). The authors reported a reduction of Myc protein levels but no reduction of *MYC* mRNA levels in P493-6 cells upon disruption of Myc-Max PPI with compound, attributed for the first time to a novel mechanism that involves destabilization, ubiquitination, and degradation of Myc. Elegantly, Choi et al. [117] showed that sAJM589 facilitated Myc turnover reducing Myc protein half-life by ~2-fold, after de novo protein synthesis was blocked by cycloheximide. Furthermore, when additional treatment with the proteasome inhibitor MG-132, which prevented the proteasome-mediated protein degradation, the increased turnover of Myc protein upon treatment with sAJM589 promoted the accumulation of ubiquitin-conjugated Myc in P493-6 cells. Thus, sAJM589 facilitated Myc ubiquitination by inhibiting Myc-Max binding and freeing the Myc C-terminal region for E3 ubiquitin ligase binding, resulting in its degradation by the proteasome. While Choi et al. [117] suggested that sAJM589 may provide a basis for the development of potential inhibitors of Myc-dependent cell growth, initial chemical optimization revealed a narrow structure-activity relationship (SAR) with sAJM589, showing superior efficacy in both PCA and cell proliferation assays.

In one of the most recent reports, Castell et al. [118] (2018) aimed at targeting the Myc-Max PPI and identified a novel small molecule inhibitor, MYCMI-6 (NSC354961), from a library of 1990 compounds from NCI/DTP Open Chemical Repository using a cell-based Bimolecular Fluorescence Complementation (BiFC) assay (Figure 7). MYCMI-6 exhibited strong selective inhibition of both c-Myc-Max and N-Myc-Max PPI in vitro at single-digit micromolar concentrations. Thoroughly, the authors demonstrated that MYCMI-6 had no effect on Mxd1 (Mad1)-Max dimerization and on Jun/Fos. Thus, among all Myc inhibitors, only MYCMI-6 and earlier 10058-F5 and 10074-G5 have been shown to be selective for Myc-Max when compared to Mad-Max. Moreover, MYCMI-6 blocked Myc-driven transcription and bound selectively to the Myc bHLHLZ domain with a *K*_D_ of 1.6 μM, as measured by the surface plasmon resonance (SPR) assay. Furthermore, MYCMI-6 inhibited tumor cell growth in a Myc-dependent manner at an IC_50_ concentration as low as 0.5 μM, but it was not cytotoxic to normal human cells. Perhaps, the greatest merit of the Castell et al. [118] study is the demonstration of the effects of MYCMI-6 administration by daily intraperitoneal injection at a dose of 20 mg/kg in vivo, where MYCMI-6 induced massive apoptosis and reduced tumor cell proliferation, tumor microvasculature density, and N-Myc-Max interaction in an N-Myc-dependent mouse xenograft tumor model based on human N-Myc amplified SK-N-DZ neuroblastoma cells, without causing severe side effects. MYCMI-6 treatment was well tolerated in mice with only slight and temporal effects on body weight.

#### 3.1.2. Direct Inhibition of Myc-Max Interaction with DNA

**Figure 8:**
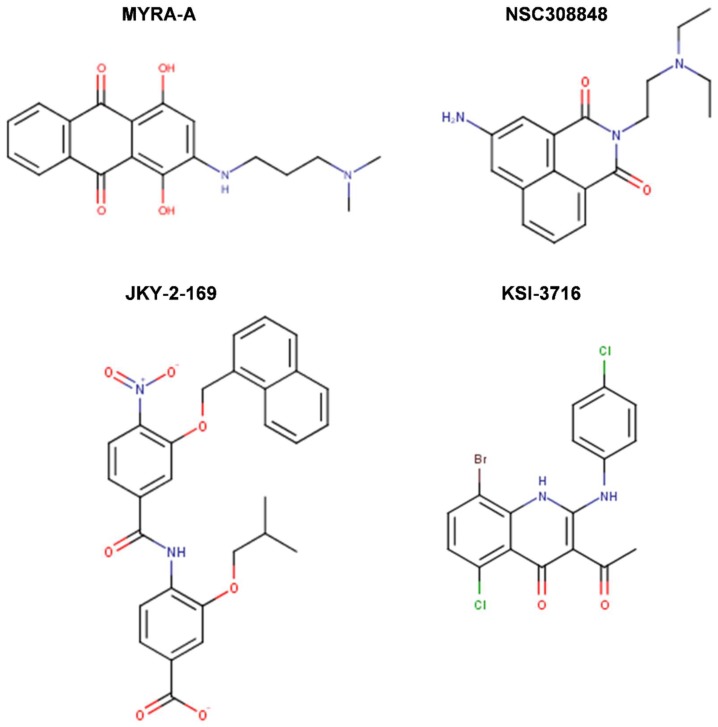
Chemical structures of MYRA-A, NSC308848, JKY-2-169, and KSI-3716 inhibitors of Myc-Max binding to DNA identified through high-throughput screening or specifically engineered to disrupt protein-DNA interactions. See text for details.

A number of studies aimed to specifically target Myc-Max binding to DNA. The developed Myc-Max/DNA disruptors include: natural-occurring compounds, such as celastrol and celastrol-inspired triterpenoids [119]; synthetic α-mimetics, such as JKY-2-169 intentionally engineered to recognize the structurally ordered Myc and hence to disrupt Myc-Max/DNA binding [120]; small molecule inhibitors (such as the Myc-pathway response agent (MYRA)-A and NSC308848) that selectively target the DNA-binding domain (DBD) of Myc-Max [121,122]; and, KSI-3716, which also blocks Myc-Max binding to DNA [123,124] (Figure 8).

Aiming at identifying a Myc-targeting therapy that could specifically affect cells with deregulated Myc in vivo, Mo et al. (2006) [121] performed a cellular screen of 1990 NCI compounds to identify those, which preferentially inhibit the proliferation of tumor cells with high Myc expression, but do not affect systems with low Myc levels. High Myc expression was obtained by using cells with inducible Myc expression (i.e., mouse fibroblasts with a tetracycline-inducible Myc transgene (Tet-Myc) treated with doxycycline in a dose- and time-dependent manner). This screen identified two compounds, known as Myc-pathway response agents, MYRA-A (NSC339585) and MYRA-B (NSC45641), which promote apoptosis in a Myc-dependent manner and inhibit Myc dependent transformation. The Myc-dependent effects of these compounds were further verified in rat fibroblasts (Rat-1 cells). MYRAs effect on cell viability was more significant in rat cells with Myc overexpression (HOmyc3) as compared with cells with wild-type Myc (TGR-1), whereas Myc-null cells (HO15.19) were highly resistant to the compounds. Importantly, MYRAs did not disrupt the dimerization of Myc-Max; instead, MYRA-A interfered with the binding of Myc-Max to DNA. Unfortunately, MYRA-A also interfered with the DNA binding of Mnt-Max and Max-Max complexes, perhaps unsurprisingly, since Mnt and Myc recognize the same DNA-binding sites and regulate an overlapping set of target genes in vivo [121]. However, MYRA-A could discriminate Myc network members from other E-box-binding proteins, as it did not affect the DNA-binding activity of the E-box binding upstream stimulatory factor, USF. MYRA-A is structurally similar to anthracyclines, but, unlike them, does not function as a DNA intercalator (i.e., as a topoisomerase II poison) since it had no effect on USF DNA-binding. All things considered, the Mo et al. [121] report was the first to demonstrate, through MYRA-A identification, the preferential inhibition of Myc overexpression combined with selective effects by induced apoptosis in a Myc-dependent manner rather than proliferative arrest. Moreover, mechanistically, MYRA-A blocked Myc-Max/DNA interactions without interfering with protein–protein interactions perhaps by sufficiently distorting the Myc-Max heterodimer rigid structure to cause a loss of DNA binding without altering the Myc-Max interactions enough to promote the dimer dissociation [121].

In a parallel study, Mo et al. (2006) [122] showed that MYRA-A also exhibits prominent effects on N-Myc overexpressing neuroblastoma cells. Moreover, they have identified a third compound, NSC308848, which also induced apoptosis in Myc-overexpressing lines (Figure 8). In contrast to the MYRAs action, treatment with NSC308848 resulted in decreased Myc protein levels and gave rise to inhibitory effects not only on Myc but also on other transcription factors, including p53. NSC308848 treatment decreased the levels of Myc in p493 B cells and in HL-60 cells, and it resulted in a dose-dependent reduction of N-Myc in Tet21N cells, while neither MYRA-A nor MYRA-B had any significant effects on the protein levels of N-Myc or c-Myc. Thus, the mechanism of action of NSC308848 differs from that of MYRA-A and MYRA-B. It is not known, however, whether NSC308848 affects Myc protein levels by interfering with transcription or by enhancing protein degradation. Mo et al.’s [121,122] findings suggest that the three small molecules can elicit a similar biological response by interfering with the Myc pathway at different levels: all three compounds induced apoptosis in Myc overexpressing cells, albeit with different mechanisms of action [121,122].

In an attempt to overcome the lack of selectivity of the earlier identified Myc inhibitors, Jung et al. (2015) [120] further designed synthetic α-mimetics specifically engineered to recognize ordered Myc in its helical form. They synthesized biphenyl based compounds that preserved the hydrophobic core and electron-rich peripheries of earlier compounds (i.e., 10074-G5 and derivatives), which were further intended to recognize a hydrophobic domain of helical Myc flanked by arginine and other polar residues that are responsible for the formation of a rigid tertiary structure upon dimerization with Max. EMSA has been used for the initial in vitro screen, followed by NMR spectroscopy and SPR biophysical assay to generate further evidence of direct binding and impairment of protein-DNA interactions. The best synthetic compound, JKY-2-169 perturbed Myc-Max binding to the canonical E-box DNA sequence without causing protein–protein dissociation in co-IP. In addition, JKY-2-169 inhibited cell proliferation of Myc overexpressing HL-60 and Daudi cells, with IC_50_s of 20 and 9.5 μM, respectively, promoted G0/G1 cell cycle arrest and the accumulation of neutral lipids. Nonetheless, compared to earlier derivatives, its K_D_ value was not significantly improved (~13 μM obtained by NMR, 10 μM in SPR) and the specificity was not enhanced either (similar activity was observed in non-cancer cells lacking Myc overexpression). The sensitivity to JKY-2-169 growth inhibition of U266 multiple myeloma (MM) cell line (IC_50_ of 46 μM), which expresses L-Myc instead of c-Myc and it was the least susceptible among MM cell lines to 10058-F4 growth inhibition (IC_50_ ~100 μM), as well as the sensitivity of HO15.19 Myc-null cells comparable to that of TGR1 cells, with IC_50_s of 20 and 14 μM, respectively, implied that JKY-2-169 had additional off-target effects and/or nonspecific toxicities. At the time of their reporting, Jung et al. [120] planned to continue the work with a focus on the determination of the metabolic stability of JKY-2-169, which retains the nitro moiety of 10074-G5 responsible for the compound’s short half-life due to the rapid metabolism to toxic hydroxylamino and other inactive amino derivatives.

In other study, Jeong et al. (2010) [123] screened with EMSA a library of 6,480 small molecules from Korea Chemical Bank and identified five compounds that blocked Myc-Max binding to DNA, with low IC_50_s of 0.58 μM for KSI-2826, 0.50 μM for FBN-1503, 2.0 μM for KSI-1449, 2.6 μM for KSI-2303, and 0.86 μM for KSI-3716. These chemicals potently suppressed Myc-dependent proliferation and induced apoptosis of HL-60 leukemia cells via G0/G1 cell cycle arrest without altering the expression level of Myc in differentiated HL-60 cells. As with the case of KJ-Pyr-9, this study did not show whether KSI-3716 inhibited Myc-Max interaction in vivo. In a follow-up study, Jeong et al. (2014) [124] showed that KSI-3716 blocked Myc-Max binding to target gene promoters and decreased Myc-mediated transcriptional activity at concentrations as low as 1 μM, as well as the expression of target genes, including *cyclin D2*, *CDK4*, and *hTERT*. KSI-3716 exerted cytotoxic effects on bladder cancer cells by inducing cell cycle arrest and apoptosis. Notably, KSI-3716 demonstrated significant growth inhibition of tumors intravesically instilled into the bladder in murine orthotopic xenograft models. With the dose of 5 mg/kg administered twice weekly for three weeks the compound demonstrated minimal systemic organ toxicity [124]. Thus, Jeong et al. [124] suggested further development of KSI-3716 as an intravesical chemotherapy agent for bladder cancer, given that KSI-3716 has several physicochemical characteristics that are suitable for such therapy. These included its drug-like molecular weight and octanol-water partition coefficient (logP), small polar surface area (PSA), and a lack of enzymatically cleavable chemical bonds (i.e., amide or ester bonds) that may confer KSI-3716 a high cell permeability, a relatively low diffusion rate in blood and other tissues, as well as an adequate intracellular concentration once absorbed by bladder and before excretion, and unlikely systemic toxicity due to negligible diffusion in the whole blood, according to its logS (i.e., low solubility). A subsequent report by Seo et al. (2014) [125] outlined that KSI-3716 had significant activity, even against gemcitabine-resistant tumors. Gemcitabine, a synthetic pyrimidine nucleoside analog, is recognized as one of the most effective first- and second-line chemotherapies, as a single agent or combination, against metastatic bladder cancer, however multidrug resistance eventually emerges. Notably, KSI-3716 suppressed gemcitabine-resistant xenograft tumors (KU19-19/GEM) that formed in the presence of as little as 2 mg/kg gemcitabine, and therapeutic potency was augmented by sequential addition of gemcitabine and KSI-3716, suggesting that the sequential combination treatment may benefit bladder cancer patients [125].

#### 3.1.3. Computational Approaches toward Myc-Max Inhibition

Novel small molecule inhibitors have been lately generated for a broad diversity of biological targets, as significant advances have been made in computational drug design techniques, such as virtual screening (VS) and molecular dynamics (MD) simulations, as well as increased access to large chemical repositories and the availability of crystallographic data.

To maximize the success of structure-based drug discovery (SBDD), cutting-edge MD simulations have recently been employed, for instance, as promising computational techniques to generate heterogeneous structural ensembles of multiple intrinsically disordered protein conformations that could be simultaneously used in VS, more specifically in what is known as “ensemble or multi-conformational docking” [126].

**Figure 9:**
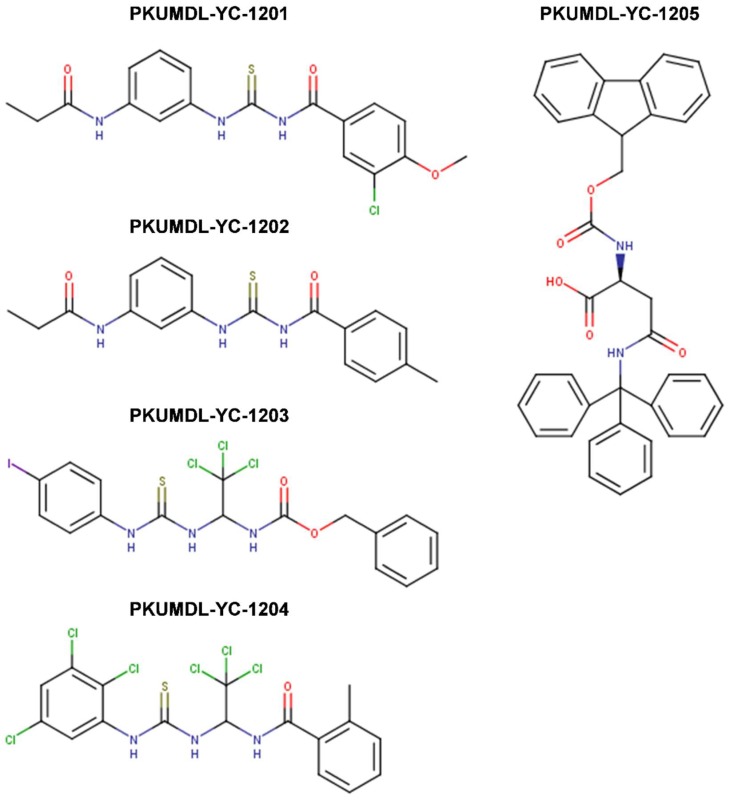
Chemical structures of PKUMDL-YC-1201 to -1205 compounds that disrupt Myc-Max dimerization identified through virtual screening (VS) multi-conformational docking against a reference ensemble of Myc disordered conformations generated through molecular dynamics (MD) simulations. Details are provided in the text.

In 2016, Yu et al. [25] provided a first successful example of a general computational approach involving comprehensive MD conformational sampling of an intrinsically disordered region of Myc, which produced an ensemble of representative conformations used for in silico binding site identification and “multi-conformational” molecular docking. Docking score analysis of the VS ensemble and “multi-conformational-affinity” compound selection were further employed to identify novel compounds. Thus, a total of 201,939 compounds from the SPECS and DCSD libraries, as well as a number of selected analogs of 10074-A4, were used for VS using the Glide docking program in standard precision (SP) mode [127,128]. The top 5% of docked compounds for different cavities identified on distinct Myc conformations were chosen for manual inspection, and 250 compounds from vendor libraries and 23 analogs of 10074-A4 were selected for experimental testing. Two classes of compounds were selected: (1) “high-conformational-specificity” compounds where the best docking score among the three identified cavities was less than −6 and the other two were greater than −4, and (2) “multi-conformational-affinity” compounds where the differences of the three scores were less than 2 and at least one of the three docking scores was less than −5. Out of 273 compounds, seven actives emerged PKUMDL-YC-1101, -1201–1205, and -1301), all binding the disordered Myc with different affinities, as determined by SPR with C-terminal bHLHLZ biotinylated Myc immobilized on the chip (K_D_s of 0.28, 17.2, 32, 0.55, 18 μM for 1101, 1201, 1203, 1204, and 1205, respectively, better than 36.3 μM for the 10074-A4 control). Four of the active compounds blocked Myc function in cell, in that they inhibited the growth of the Myc-overexpressing HL-60 cells in an MTT assay (EC_50_s of 6.9, 8.8, and 40 μM for 1203, 1204, and 1205, respectively, relative to 15.1 μM for 10074-A4 control), affected cell cycle progression in a dose-dependent manner increasing the S-phase while decreasing the G2/M phase, suggesting that the observed effect on viability was not due to cytotoxity. In addition, the four compounds blocked Myc-mediated transcriptional activity in HL-60 cells, as demonstrated in qRT-PCR showing a reduction in *cyclin D2* and *CDK4* downstream Myc regulated genes, confirming that the cell cycle progression arrest was caused by Myc inhibition. Apart from PKUMDL-YC-1101, all six actives from docking as well as 10074-A4 were “multi-conformational-affinity” compounds. Compounds PKUMDL-YC-1201, PKUMDL-YC-1202, PKUMDL-YC-1203, and PKUMDL-YC-1204 shared a common thiourea structure (Figure 9). Even though PKUMDL-YC-1203 and PKUMDL-YC-1204 had better experimental profiles, they were less soluble in water, prompting the authors to further investigate PKUMDL-YC-1205, an analog of 10074-A4, instead.

In an SPR competitive assay with GST-tagged Max protein immobilized on a chip, PKUMDL-YC-1205 abolished Myc binding to Max in the dissociation curves at concentrations in the 100–800 μM range. Chemical cross-linking and anti-Max Western blotting experiments showed disruption of Max-Max/Myc-Max dimerization equilibrium. Treatment with PKUMDL-YC-1205 decreased the Myc-Max heterodimer ratio, consequently leading to an increased Max homodimer ratio. As the parental compound, PKUMDL-YC-1205 is a disruptor of Myc-Max protein–protein interactions. Five independent 100-nanosecond MD simulations in explicit solvent were then performed, using the Amber molecular dynamics package [129], for each of the following: Myc/PKUMDL-YC-1205, Myc/10074-A4, and Myc/AJ-292/41944612 (an inactive compound) docked complexes as initial structures. Analysis of the trajectories showed that PKUMDL-YC-1205 and 10074-A4, which were “multi-conformational-affinity” compounds, had longer binding times than the non-active and “high-conformational-specificity” compound during the simulation course. Yu et al. [25] provided a useful strategy for SBDD targeting intrinsically disordered proteins (IDPs) while also suggesting a tendency for IDPs to bind to “multi-conformational-affinity” compounds–compounds that bind to various groups of conformations with similar affinity, instead of “high-conformational-specificity” ones—with high affinity to one class of conformation but very low affinity to others.

**Figure 10:**
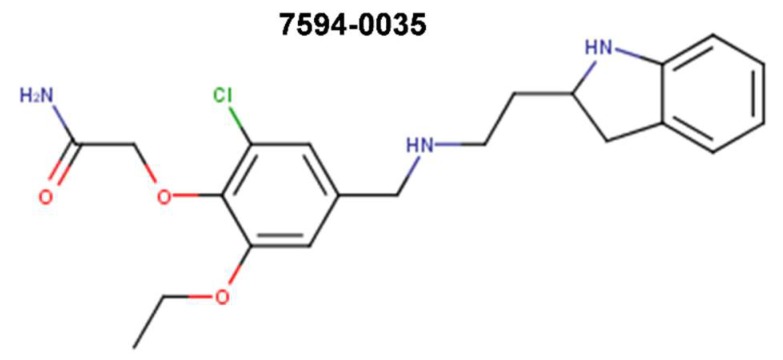
Chemical structure of 7594-0035 Myc-Max inhibitor identified in silico utilizing the Myc-Max 1NKP X-ray structure, yet targeting a previously reported disordered binding region for 10074-G5 but in ordered form. See Section 3.1.4 for further details.

In 2018, Yao et al. [130] reported yet another novel inhibitor, 7594-0035, specifically targeting Myc for the potential treatment of relapsed/refractory multiple myeloma (MM), in which frontline therapy-resistance has been associated with Myc [131,132]. Compound 7594-0035 has been identified from the ChemDiv database, a commercially available small molecules repository containing more than one-million entries, through VS and utilizing the published crystal structure of Myc-Max complexed with DNA [75] (PDB ID: 1NKP) (Figure 10). Interestingly, although the group considered the ordered dimer structure for virtual screening, no attempts have been made to identify pockets on the dimer surface through computational means. Instead, the investigators considered as the docking site the previously described disordered region bound by the 10074-G5 inhibitor (see Section 3.1.4), but in ordered form, due to the fact that the 1NKP X-ray structure of the Myc-Max complex lacks a bound small molecule ligand. For that matter, and importantly, no ligated X-ray structure of Myc-Max exists. The Surflex molecular docking [133] module in the Sybyl-X2.1 molecular modeling and simulation suite [134] was used for virtual screening. 200 ChemDiv compounds were purchased after two rounds of screening, one intended to accelerate the docking process by reducing the number of conformers and rotatable bonds and a second one to re-screen the top 1% compounds using default parameters at the selected site. Compound 7594-0035 showed a high docking score (<−6), and following experimental validation showed potent Myc inhibitory activity. This compound inhibited the proliferation of RPMI-8226 and U266 multiple myeloma cells in vitro at concentrations in the 20–40 μM range, induced cell cycle G2 phase arrest and triggered their apoptosis by disturbing the stability of the Myc protein, while Myc mRNA levels were unaffected. It has been demonstrated that treatment with 7594-0035 resulted in rapid degradation of Myc protein after the inhibition of protein synthesis with cycloheximide and the proteasome inhibition with MG132. Moreover, it overcame drug-resistance to bortezomib also a proteasome inhibitor that is considered a breakthrough in the treatment of MM [135], and increased the killing effect of MM cells in combination with bortezomib. 7594–0035 also decreased primary tumor growth in vivo (SCID mouse xenograft model subcutaneously injected with RPMI-8226 cells). As it showed a significant anti-tumor effect on MM cells, this compound may be a promising therapeutic agent for MM.

**Figure 11:**
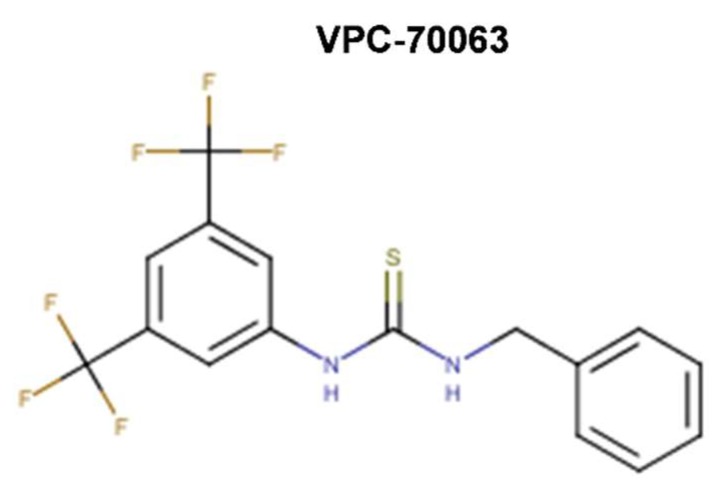
Chemical structure of VPC-70063 identified in silico by screening of the largest purchasable chemical space of the ZINC database that targets a novel binding site located at the Myc-Max/DNA interface of the 1NKP Myc-Max X-ray structure. See Section 3.1.4 for further details.

Recently, our group [136] applied a rational computer-aided drug discovery (CADD) approach to identify Myc-Max inhibitors as potential therapeutics for prostate cancer (PCa). In PCa, the three Myc paralogs are most frequently amplified and implicated in the pathogenesis and progression across its full spectrum, from localized adenocarcinoma (L-Myc) to the most advanced and treatment-resistant subtypes—castration-resistant (c-Myc) and its neuroendocrine phenotype (N-Myc) [137,138,139,140,141,142,143,144]. We utilized the X-ray structure of Myc-Max bound to its DNA recognition sequence [75] (PDB ID: 1NKP) and identified three plausible binding sites on the ordered dimer interface (see Section 3.1.4 for a detailed description), out of which the site located at the dimer/DNA interface, being the top-ranked pocket, was prioritized for targeting with small-molecules. We conducted a large-scale virtual screening implementing structure-based methodologies, including molecular docking and pharmacophore modeling against 4.7 million drug-like compounds from the ~6 million purchasable chemical space of the ZINC12 molecular database [145,146] reduced by physicochemical properties, including charge, number of rotatable bonds, and number of rings. Docking poses, obtained using Glide (Maestro 9.3 suite, Schrödinger LLC) software [127,128,147] with SP mode and default parameters, were filtered down by their binding affinity docking scores using a −5.5 kcal/mol cutoff, ligand efficiency, subsequent pharmacophore feature-matching, and satisfaction of additional favorable interactions within the site. 69 chemicals were purchased for experimental testing from the millions at the start. From 10 compounds that showed greater than 50% inhibition of Myc-Max transcriptional activity in LNCaP PCa cells, as determined by a commercially available luciferase-based Myc reporter assay that was utilized as a primary screen, VPC-70063 (Figure 11) showed the best inhibition of Myc activity with an IC_50_ of 8.9 μM and it was prioritized for further in vitro mechanistic evaluation. VPC-70063 affected the Myc-Max AR-V7/UBE2C downstream pathway showing the highest reduction of levels of the constitutively active ligand-independent androgen receptor splice variant AR-V7 that promotes advanced castration-resistant prostate cancer [148,149], in androgen-deprived 22rv1 cells. AR-V7 reduction in 22rv1 cells due to Myc-Max inhibition was confirmed by Western blot analysis. VPC-70063 inhibited the proliferation of Myc-positive LNCaP cells with an IC_50_ of 2.5 μM with no significant effect on the HO15.19 Myc-null cell line at a 3 μM concentration where the effect on viability was observed in LNCaP cells, indicating a specific on-target inhibitory effect. VPC-70063 induced apoptosis in LNCaP cells indicated by poly (ADP-ribose) polymerase (PARP) cleavage, suggesting a specific mechanistic effect and no general toxicity. Moreover, VPC-70063 blocked Myc-Max binding to DNA, as quantified by biolayer interferometry (BLI) (with biotinylated E-box DNA sequence immobilized on the sensor) in a dose-dependent manner. In addition, treatment with 70063 resulted in a reduction of Myc protein levels in LNCaP cells. The development of VPC-70063 is ongoing to improve its potency and to remove the minimal toxicity observed at higher concentrations in the Myc-null cell line. The in silico hit-to-lead optimization protocol involving both structure- and ligand-based modeling techniques and consensus scoring computations has been fully described in our study [136]. A number of improved analogs have already been identified (unpublished data) and, along with the parental compound, are currently assessed for their metabolic stability, solubility, permeability, and undesired drug-drug interactions. Dependent on the suitability of their pharmacokinetic profiles, additional medicinal chemistry efforts will be undertaken to develop lead compounds for future in vivo efficacy and safety studies.

An inverse strategy to reduce Myc-Max activity pursed by computational techniques is minimizing the availability of Max by stabilizing Max-Max repressive homodimers competing with Myc-Max at the same E-boxes. It has been rationalized that given the rather unique packing defect of the Max homodimer (described in Section 2.3) that makes Max-Max less stable than Myc-Max or other heterodimers of the extended network, specific small molecule stabilizers of Max-Max interactions could potentiate the downregulation of the network [75,150].

**Figure 12:**
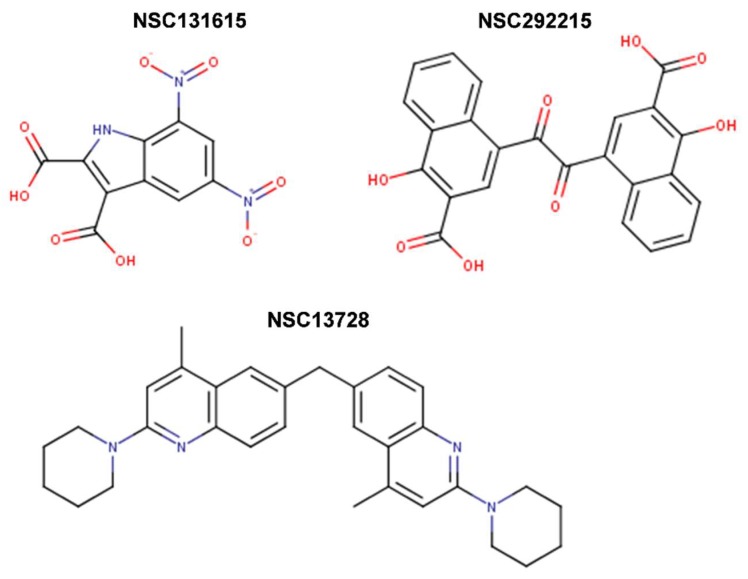
Chemical structures of representative Max-Max stabilizers that alter Myc-Max function by blocking binding to equivalent DNA binding sites. See text for details.

In 2009, Jiang et al. [150] isolated Max-Max stabilizers by applying “blind docking”, a virtual screening technique for sampling large regions of protein complexes, and three-dimensional clustering analysis to identify specific binding pockets for small-molecule interactors, constituting the first computational study to screen a medium-sized database of compounds over entire structurally ordered dimers. The AutoDock 3.0.5 software suite [151] was used to find the best fit chemicals out of 1668 selected from 140,000 NCI compounds for VS targeting both Myc-Max [75] and Max-Max [74] X-ray dimer structures (PDB IDs: 1NKP and 1AN2). Docked poses were iteratively clustered, based on their predicted binding location, lowest binding scores, and inclusion in a 10 Å distance from center of mass. Three main clusters emerged that contained 85% of docked compounds, including those with lowest predicted binding energies. The compounds in each cluster exhibited similar trends in their chemical properties. VS results for the Max homodimer and the Myc-Max heterodimer were similar for cluster 1, which contained 456 compounds total, including those with the lowest predicted binding energies from the entire NCI set, and generally displaying an abundance of negatively charged atoms or a high density of hydrogen bonding atoms. A representative compound for cluster 1 is NSC131615 (Figure 12).

Cluster 2, the smallest of the three bundles, seconded cluster 1 in terms of predicted binding affinities. While similarly charged, the 90 compounds comprising the second cluster, representative of which is compound NSC292215 (Figure 12), were more hydrophobic. Although the majority of selected compounds grouped in cluster 3 (863 chemicals total), their strength of binding was weaker relative to those fitting clusters 1 and 2. The chemical structures for cluster 3 compounds were characterized by the presence of one or more rigid, flat surfaces, and a lack of a strong negative charge. 68 unique compounds (40 from the Max-Max VS, 40 from the Myc-Max VS, and 12 removed for being common to both sets) representing the top docking solutions from the three clusters with predicted binding energies from −11.3 to −8.4 kcal/mol were requested from NCI for further experimental screening in a primary FRET assay. FRET analyses correlating with the molecular docking results showed that 85% of the compounds of cluster 3 were specific for the Max homodimer, as compared to 67% for cluster 2 and only 13% for cluster 1. Compound NSC13728, the representative of cluster 3, the most effective one, was thus selected for further characterization (Figure 12). In vitro binding assays showed that NSC13728 enhanced Max-Max homodimerization, while it also interfered with Myc-Max heterodimerization (co-IP, ELISA, SPR, and ultracentrifugation analysis). EMSA analysis showed that NSC13728 that targets the intersection of the LZ and HLH regions did not significantly affect DNA binding of the Myc-Max heterodimer or that of the Max-Max homodimer in contrast to the compounds of cluster 1 and 2, predicted to interact with the basic DNA binding region of Myc or Max, which decreased DNA binding significantly. Nonetheless, NSC13728 interfered with Myc-mediated oncogenic transformation of CEF with an IC_50_ of 3 μM, but not with that induced by the Src oncoprotein. NSC13728 inhibited the growth of the MCF7-35IM breast cancer cell line that carries an inducible Myc transgene, and when used in combination with the estrogen antagonist ICI 182,780 that inhibits endogenous Myc expression, an additive reduction was observed. NSC13728 also attenuated Myc-dependent transcription at concentrations in the 2.5–10 μM range, as a result of the stabilization of the Max-Max homodimer. Although limited in the chemical space that was employed for virtual screening, this study nonetheless provided proof-of-concept that ordered dimer structures are more promising in silico docking targets for small-molecule disruptors over the disordered monomeric forms.

#### 3.1.4. Binding Sites for Myc-Max Small-Molecule Inhibitors

Despite the growing number of novel Myc-Max inhibitors, there is a paucity of information with respect to their precise binding mode. As a matter of fact, binding sites have been determined solely for a handful of inhibitors.

**Figure 13:**
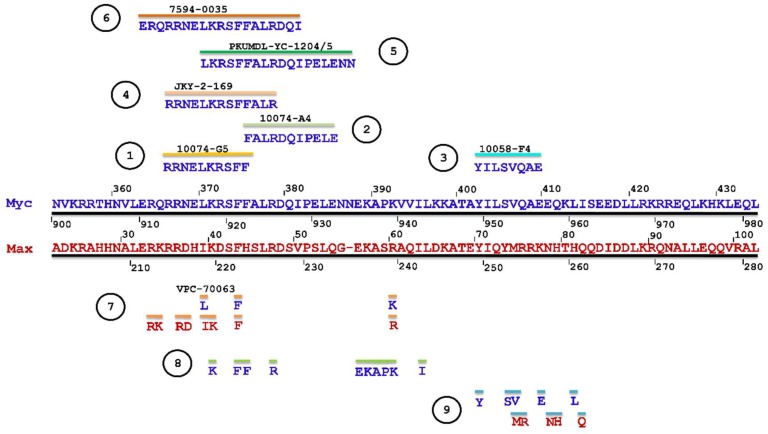
Binding sites for structurally diverse Myc-Max inhibitors mapped along Myc and Max protein sequences. Residue numbering above a protein sequence follows the convention for individual monomers, while that bellow follows the numbering in the Myc-Max X-ray structure. Binding sites described in the text are numbered and illustrated by circle-enclosed digits. The coloring scheme suggests three major druggable sites on Myc, both in disordered monomeric form or in the functional ordered form of the Myc-Max heterodimer.

Early binding sites for compounds 10058-F4 and 10074-G5, Myc_402–412_ and Myc_363–381_ (Figure 13), respectively, were identified through point mutations and truncations of synthetic peptides of the Myc DNA-binding and dimerization domains, followed by circular dichroism (CD) and NMR spectroscopy [84]. Direct binding to purified recombinant Myc bHLHLZ domain, Myc_353–437_, was monitored using a fluorescent polarization assay to capitalize on the intrinsic fluorescence exhibited by 10058-F4 and 10074-G5. Binding of 10058-F4 was impaired by mutations of residues at the interface between H2 and LZ regions: Leu404Pro, Gln407Lys, and Val406Ala-Glu409Val and by deletion of the LZ region Myc_406–437_ (Figure 13). By contrast, binding of 10074-G5 was enhanced by amino acid substitutions between the basic region and H1: Arg367Gly and Glu369Lys-Leu370Pro and eliminated by truncations Myc_370–409_ and Myc_400–439_. Other ~20 mutations did not have a substantial effect on binding affinity.

Flexible molecular docking of 10058-F4 into Myc_402–412_ peptide segment showed that the inhibitor was located at the center of a C-shaped cavity that formed upon the binding of compound to the peptide segment, in an orientation that allowed for intramolecular hydrophobic interactions to take place between its aromatic ring and the ethyl tail and intermolecular hydrophobic interactions with the N-terminal hydrophobic aromatic and aliphatic side chains of the peptide: primarily, Tyr402, Leu404, and Val406, but also Ile403 and Ala408. Additionally, the carbonyl oxygen of 10058-F4 was within H-bonding distance with Ser405 and Gln407 side chains. This model matched independent NOESY results that indicated the formation of a similar hydrophobic cluster.

The model of 10074-G5 binding to Myc_363–381_ revealed that the inhibitor docked to a pocket that was dynamically formed by a sharp twist in an N-terminal helical segment extending from Leu370 to Arg378, in agreement with independently generated NOESY data. In its binding pose, the ortho-biphenyl moiety of 10074-G5 was in close proximity to the aromatic ring of Phe375, while the furazan and nitro electron-rich moieties interacted with the positively charged Arg366-367. Binding of 10058-F4 and 10074-G5 to the disorder Myc induced global conformational changes that abrogated Myc-Max interactions. The small molecule-induced bound structures nevertheless remained disordered and differed from the ordered structure induced by Max in the bHLHLZ of Myc. Free and bound structures of the above-mentioned Myc peptides were modeled using the PREDITOR web server [152] based on dihedral constraints that were obtained from observed NMR chemical shifts, and further energy minimized using CHARMM27 parameters [153]. Docking simulations were then performed between the optimized bound structures and respective inhibitors using the AutoDock Lamarckian genetic algorithm (LGA) to obtain the final models representing the most reasonable conformations out of the dynamic ensemble of each complex [84,151]. To further assess the potential determinants of binding specificity of these small molecules to the nonconventional, flexible binding sites that were identified in Myc, a comparison between the Myc bHLHLZ sequence and those of related Max and Mad bHLHLZ proteins have been performed to reveal several conservation anomalies where sequence conservation was observed to some extent between Max and Mad but not Myc [84]. 10058-F4 and 10074-G5 have been previously shown to selectively inhibit Myc-Max, but not Mad-Max by Yin et al. [99]. Out of 22 anomalies that were scattered throughout the Myc_353–437_ sequence, four occurred within the 10074-G5 binding site (Asn368, Glu369, Phe375, and Ala376), five occurred within 10058-F4 binding site (Leu404, Ser405, Val406, Gln407, and Ala408; for E409 there is no consensus at all), and three (the already mentioned Phe375 and Ala376, and also Glu383) occurred within the binding site of yet another inhibitor identified earlier by Yin et al. [99], 10074-A4, a non-fluorescent compound (discussed further below). The conservation anomalies are indicated with a star (*) below the multiple sequence alignment in Figure 4.

Moreover, the early identified binding regions Myc_402–412_ and Myc_363–381_ (Figure 13) for compounds 10058-F4 and 10074-G5, contained two of the three clusters of hydrophobic residues that were found in Myc bHLHLZ: 374-Phe-Phe-Ala-Leu-377 and 401-Ala-Tyr-Ile-Leu-404 [84], which were more hydrophobic than the corresponding sequences of other bHLHLZ proteins: Leu-Glu-Lys-Leu and Leu/Met-His-Ile-Lys/Gln in Mad; Leu-Glu-Arg-Leu and Ala-His-Ile-Lys in Mxi; and, Phe-His-Ser-Leu and Glu-Tyr-Ile-Gln in Max, respectively. Follis et al. [84] findings highlighted the importance of high hydrophobic content and low disorder propensity as properties that may be prevalent in IDPs and most relevant for targeting protein–protein interactions of IDPs with small-molecules, setting a first practical example. In 2014, 10074-G5 and 10058-F4 binding sites were confirmed, and importantly, were established on both c- and N-Myc in their monomeric disordered form by Muller et al. [154].

Subsequent CD and NMR spectroscopy experiments that were conducted by Hammoudeh et al. (2009) [155] showed that compounds 10058-F4, 10074-A4, and 10074-G5 bind specifically the Myc monomer at three simultaneous and independent binding sites (Figure 13). These sites consisted of contiguous stretched of amino acids: Myc_366–375_ within the Myc_363–381_ peptide for 10074-G5 (site 1 in Figure 13), Myc_375–385_ within the Myc_370–409_ peptide for 10074-A4 (site 2 in Figure 13), and Myc_402–409_ within the Myc_402–412_ peptide for 10058-F4 (site 3 in Figure 13), which matched the highest chemical-shift peaks also found on full-length Myc_353–437_. The reported models of Myc-bound by compounds were generated based on the average of multiple dynamic structures of the intrinsically disordered domain of Myc, as previously reported by the group [84]. The affinity of 10058-F4 for its cognate binding site was K_D_ = 5.3 μM but more than doubled when the minimal peptide binding site (residues 402–412) was extended to include the entire bHLHLZ domain (K_D_ = 13 μM). A similar increase in affinity also occurred in the case of 100F4-G5—from K_D_ = 2.8 μM to 4.4 μM. This suggested that the specificity of site recognition by these small molecules as well as the fine-tuning of binding affinities arose from residues that extended to a certain degree beyond the minimal binding sites defined previously by synthetic peptides [84].

Competition titrations using fluorescent polarization performed by Hammoudeh et al. [155] further showed that out of the seven inhibitors that were originally identified by Yin et al. [99], compounds 10031-B8, 10075-G5, and 10009-G9 also bound at the 10058-F4 site in the H2-LZ region (site 3 Figure 13) but with three- to eight-fold lower affinity than 10058-F4, while compound 10050-C10 (the largest one) bound at the 10074-G5 site in the basic-H1 region (site 1 Figure 13), this time with a three-fold higher affinity (*K*_D_ = 0.9 μM) than 10074-G5, indicating the plasticity of intrinsically disordered binding sites. Compound 10074-A4, unlike 10058-F4 and 10074-G5, is a non-fluorescent chemical that exists as a racemic mixture of two (R- and S-) enantiomers [99,155]. The structural features of the binding interaction between 10074-A4 and its binding site, Myc_370__–__409_ (site 2 in Figure 13) deduced by CD (*K*_D_ = 21 μM), were further characterized by NMR, which indicated that the exact location of the interaction site for 10074-G4 was immediate C-terminal to that of 10074-G5 (site 1 in Figure 13) [155]. Significant localized interactions within the H1 and loop regions, as determined by NMR backbone chemical shifts, were observed to include the predominant hydrophobic interactions between 10074-G4 and hydrophobic residues Leu377, Ile381, and Leu384 of the peptide, as well as interactions with Arg378 and Asp379. A comparison of free and bound peptide models further showed that the compound was enclosed in a cavity shaped by N-terminal residues Phe374, Phe375, Ala376, and Leu377 of the loop region, and Leu377 of the H1 helix. Docking of 10074-A4 to the bound conformation of the peptide showed that the compound was stabilized via extensive hydrophobic interactions. The docking of both enantiomers of 10074-A4 displayed a similar mode of binding and similar docking scores. Poses for both enantiomers were generally consistent with the independently generated NMR NOE data. The docking simulation provided a general understanding of the binding interaction, but it could not generate precise binding information or identification of a favored binding enantiomer [155]. As already mentioned, the group further designed the JKY-2-169 α-mimetic compound [120], the best of a series of biphenyl based compounds that were intended to specifically recognize the ordered region Myc_366–378_ that included the binding region of 10074-G5 as well as hydrophobic N-terminal binding region of 10074-A4. The binding site of JKY-2-169 was delineated by Arg372, Phe374, Leu377, and Arg378 (site 4 in Figure 13).

Nowadays, MD simulations are massively employed to study and understand the characteristics of highly dynamic conformations of IDPs, providing a detailed all-atom description of molecular interactions within a system as it evolves in time. MD is the computational method that best complements X-ray and NMR-based techniques that may lack sufficient resolution to fully investigate IDPs, for they cannot reveal the complete dynamic behavior of IDPs in atomistic details, nonetheless experimental structural data, such as NMR chemical shifts, are necessary to evaluate MD ensemble models for improved accuracy [156]. MD is markedly useful to study weak interactions between a ligand molecule and an IDP, since these interactions are often associated with rapid conformational changes [157].

In a 2012 report, Michel et al. [158], using explicit solvent enhanced sampling meta-dynamics simulations on 10058-F4, reported that 10058-F4 interacts with a Myc_402–412_ conformational ensemble at multiple different dynamically-formed pockets within the peptide segment, and that the ligand binding was driven by weak and non-specific interactions, but made preferential contacts with Tyr402, Ile403, and Leu404, which lie within the most hydrophobic segment of the entire bHLHLZ domain. In Max, such segment does not exist.

In 2013, Jin et al. [24] studied the binding characteristics of 10074-A4, the only Myc compound known insofar to bind to the loop region, by using extensive MD simulations, with both implicit and explicit solvent models, and found that 10074-A4 associated with Myc_370–409_ and behaved like a “ligand cloud” around a “protein cloud”, with distinct features from that of a non-binding negative control peptide segment, Myc_410–437_. They found that 10074-A4 bound Myc_370–409_ at different sites along Myc disordered conformations. The results that were obtained from MD simulations were consistent with the NMR data, thus providing evidence of the reliability of computational approaches. Importantly, Jin et al. [24] obtained a conformational ensemble of apo (free) and holo (bound) forms that are suitable for use as reference structures for drug design targeting Myc through SBDD approaches. Their extensive simulations consisted of 34.5 μs total time in implicit solvent with 30 REMD replicas each of 1.15 μs for four groups encompassing their own-built extended structure of the Myc_370–409_ peptide (150 ns), the apo NMR defined structure (270 ns), the structure obtained after 80-ns simulation time from the extended structure (210 ns), and the most occupied representative conformations that were previously generated by REMD (replica-exchange molecular dynamics) from the built-in extended structure (520 ns). The explicit solvent simulations were conducted for a total of 21 μs for three groups: 1 μs of seven trajectories, for each 1 apo and 2 holo for chiral forms of A4, using as starting structure the NMR refined structure, and six of the representative conformations generated by the 150-ns extended peptide REMD simulations. The negative peptide control was simulated for 80 ns in implicit solvent with the extended Myc_410–437_ peptide used as initial structure. The final structure was applied on all-atom explicit simulations, 1 μs each per 10074-A4 chiral forms.

Naturally, VS aided by MD re-emerged in 2016 as a powerful synergistic combination of computational methods for the discovery of novel inhibitors targeting representative conformations of IDPs at multiple sites. Indeed, Yu et al. [25] utilized the reference ensemble of the apo and holo conformations of the Myc_370–409_ intrinsically disordered region generated earlier [24] for in silico binding site identification using the CAVITY program [159]. Three sites were identified: two sites in the apo conformation, termed Apo1 in Myc_379–409_ and Apo2 in Myc_370–386_, and one site in the holo conformation, Holo1 in Myc_374–388_. Virtual screening was then conducted targeting the aforementioned sites that led to the discovery of the PKUMDL-YC prefixed inhibitors that are described in Section 3.1.3. Structurally, compounds PKUMDL-YC-1201, PKUMDL-YC-1202, PKUMDL-YC-1203, and PKUMDL-YC-1204 shared two common chemical features, namely thiourea and acylamino groups, which as docked engaged through their amino hydrogen atoms in H-bonding interactions with the backbone oxygen atoms of Glu383 and Asp379, respectively. These observations prompted Yu et al. [25] to conduct a structure-activity relationships (SAR) analysis to gain insights into the determinants of their binding affinity. In their docking mode, the thiourea and acylamino groups of PKUMDL-YC-1203 and PKUMDL-YC-1204 formed additional hydrogen bonds with the backbone carbonyl of Ile381. On top of these, PKUMDL-YC-1203 and PKUMDL-YC-1204 further engaged Arg378 in H-bonding interactions. PKUMDL-YC-1203 was less active than PKUMDL-YC-1204 due to electrostatic repulsion between the oxygen from the benzyloxy group of PKUMDL-YC-1203 and the backbone oxygen atom from Asp379. Because PKUMDL-YC-1203 and PKUMDL-YC-1204 were less soluble in water, a detailed binding analysis by Saturation-Transfer Difference (STD) NMR was conducted for PKUMDL-YC-1205, the analog of 10074-A4, instead. The interactions between PKUMDL-YC-1205 or 10074-A4 (the favored S enantiomer form) and Myc_370–409_ were further investigated by five independent 100-nanosecond MD simulations using the molecular docking complexes as initial complex structures. Yu et al. [25] showed that, despite the fact that the compounds were residing in their binding sites in the MD initial structures, they hovered along the Myc_370–409_ structure during the simulation course. To characterize the binding site, the interaction distances within 5 Å between the compounds and residues in Myc_370–409_ were calculated. PKUMDL-YC-1205 and 10074-A4 showed a strong tendency to bind to the N-terminal residues 370–387 and 375–385 in Myc_370–409_, respectively (site 5 in Figure 13). Particular contacts for PKUMDL-YC-1205 within the Myc_370–387_ region were modeled as: Lys371, Arg372, Ser373 (confirmed by NMR TOCSY spectrum), Phe375, Gln380, Ile381, and Pro382. Yu et al. [25] study provided a useful strategy for structure-based drug discovery targeting IDPs and proof-of-concept that “protein clouds” are druggable.

Also, mentioned earlier, is the novel Myc-Max inhibitor 7594-0035 discovered by Yao et al. [130] through docking-based virtual screening using the X-ray structure of Myc-Max complexed with DNA [75] (PDB ID: 1NKP). Interestingly, although the group considered the dimer structure for docking, no attempt has been made to identify new pockets on the dimer surface in silico. Instead, the investigators considered the previously described disordered region, Arg363-Ile381 of Myc bound by the 10074-G5 inhibitor, but in ordered form, as the site for inhibitor binding, given that there is no small-molecule ligand bound to the Myc-Max complex in the 1NKP X-ray structure. Therefore, during the preparation of the protein for docking, only the region Arg363-Ile381 of the ordered dimer was set as the docking site (site 6 in Figure 13), and the loop382-392 region was removed, because, in the stable ordered state of Myc, the loop is in close proximity to the pocket, with the Lys392 side chain inserting into the active site [130]. In its docking pose, compound 7594-0035 formed a strong H-bonding network with the side chains of residues Arg364, Arg367, and Asn368, and the main chain of residue Arg364. Apart from the polar interactions, the indole ring of compound 7594-0035 was accommodated in a hydrophobic cavity formed by Leu379, Lys371, Phe374, and Phe375. Likely both hydrogen-bonding and hydrophobic interactions played key roles in the binding of 7594-0035 to Myc [130].

**Figure 14:**
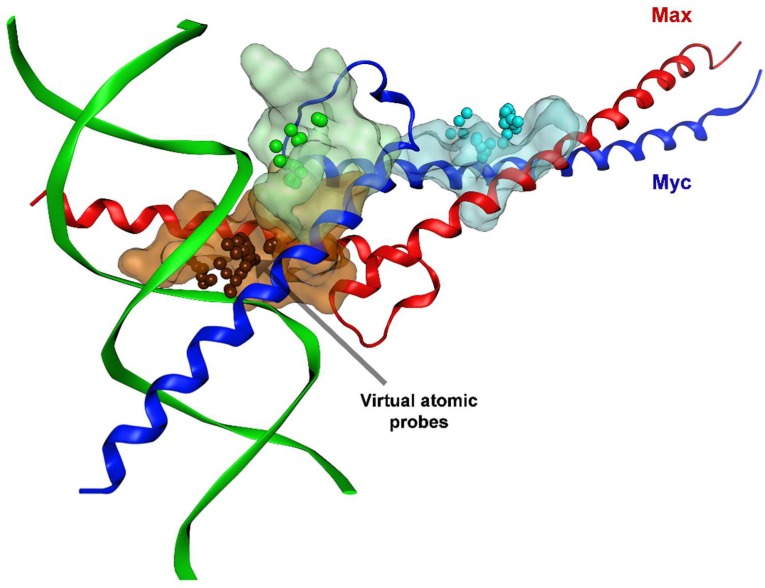
Three independent binding sites identified in silico at the Myc-Max/DNA and Myc-Max dimerization interfaces (colored meshed surfaces). The sites were identified by probing the surface with virtual atoms (colored alpha spheres within the three found pockets). The image was generated with Molecular Operating Environment (MOE). See text for details.

Independently, our group utilized the X-ray structure of the Myc-Max heterodimer bound to its DNA recognition sequence [75] (PDB ID: 1NKP) for in silico identification of plausible binding sites at the ordered dimer surface and subsequent large-scale virtual screening targeting these sites [136]. Using the Site Finder module of the Molecular Operating Environment (MOE)—a fully integrated drug discovery software platform [160], three main sites (Figure 14) were predicted as the most probable druggable pockets. The Site Finder algorithm calculates binding sites by scanning a receptor surface with virtual atoms (α spheres) of either hydrophilic or hydrophobic character that are subsequently clustered. The sites are then scored and ranked based on their size and Propensity for Ligand Binding (PLB) [161], an index accounting for amino acid composition at the receptor-ligand interacting interface. The top PLB-ranked site (site 7 in Figure 13) was situated at the Myc-Max interface with the DNA major groove between the bHLH DNA-binding regions of Myc and Max. This first site on the Myc-Max dimer structure contained highly-conserved residues from both Myc and Max monomers, and as such differed from all of the previously described sites on Myc monomer only, both in its disordered and ordered forms. Hydrophobic residues Leu917 and Phe921 from the Myc monomer and the equivalent residues Ile218 and Phe222 from Max form the hydrophobic core of this pocket. Charged residues Lys939 from Myc and Arg212, Arg215, Asp216, Lys219, and Arg239 from Max line up the pocket providing additional propensity for electrostatic interactions. Compound VPC-70063 specifically targeting the first top-ranked site was the best performing, as characterized in a panel of cell-based and cell-free assays (see Section 3.1.3). Chemically, the structure of VPC-70063 consists of two aromatic moieties at either end, a benzyl ring and a highly hydrophobic 3,5-bis(trifluoromethyl)phenyl group, which are connected by a thiourea linker. In its docking pose, VPC-70063 interacts with the backbone carbonyl of Arg215 via 2 hydrogens bonds with the two amine hydrogens of the thiourea linker. Moreover, VPC-70063 forms a large number of strong hydrophobic interactions. Its 3,5-bis(trifluoromethyl)phenyl moiety is deeply buried in the hydrophobic core of Myc-Max DBD pocket where it is stabilized by hydrophobic interactions with the aromatic side-chains of Phe921 of Myc and Phe222 of Max, as well as with the aliphatic side-chains of Leu917 and Lys939 of Myc, and Ile218 and Arg215 of Max. Furthermore, the benzyl ring of VPC-70063 forms hydrophobic interactions with the aliphatic side-chains of Arg215 and Arg212 of Max. Importantly, in the binding pose, the benzyl ring significantly overlapped with the DNA backbone.

We report that the optimization protocol described in Carabet et al. [136] study resulted in more potent analogs of VPC-70063 that maintain the hydrophobic core and H-bonding interactions, while displaying enhanced inhibitory effects on Myc function with no toxicity in prostate cancer models (unpublished data). Novel scaffolds were also identified. Furthermore, the new derivatives have similar inhibitory effects on N-Myc amplified cell lines (unpublished data), unsurprisingly since the binding site composition and chemical character is identical in both the c-Myc-Max X-ray dimer structure as well as in our N-Myc-Max homology-model built at high 0.7 Å resolution using MODELLER [162,163] with the 1NKP X-ray structure used as a template. While c-Myc- and N-Myc-Max sites at the DNA interface are conserved, the Mad-Max site (the only major identified by Site Finder on the 1NLW X-ray structure [75]) differs in amino acid composition, charge, and size, as is composed of: Arg14, Leu17, Arg18, Leu21, and Thr39 from Mad (equivalent to Arg914, Leu917, Lys918, Phe921, and Lys939 on Myc), and Arg214, Arg215, Ile218, Lys219, Phe222, and Arg239 on Max. There are more residues contributing to this site on Mad as compared to Myc, with two additional positively-charged arginine residues in the site as well as two important substitutions, one from an aromatic to an aliphatic residue at position 921 in Myc (Phe921Leu) and the other from a positively-charged to a neutral amino acid at position 939 in Myc (Lys939Thr), therefore specific inhibitors or stabilizers of the complexes could be designed. The second PLB-ranked site (site 8 in Figure 13) was predicted to contain residues in the HLH region from the ordered Myc monomer only: Lys918, Phe921, Phe922, Arg925, Glu935, Lys936, Ala937, Pro938, Lys939, and Ile942. It neighbors the first site, but it is more hydrophobic when compared to the highly-positively charged first site. Myc specific contacts in this site are Phe922, Lys939, and Pro938, which in Mad are non-conservatively substituted for a glutamic acid and two threonine residues, respectively. The third site (site 9 in Figure 13) located at the intersection of H2 and LZ regions is the smallest and the most surface-exposed pocket. It is shaped by residues Tyr949, Ser952, Val953, Glu956, and Leu960 from Myc, and Met253, Arg254, Asn257, His258, and Gln261 from Max. This is the site in which the most conservation anomalies exist with most Myc residues diverging from the consensus among bHLHLZ proteins, including Ser952 (consensus Lys), Val953 (consensus Ala), Glu956 (no consensus), and Leu960 (consensus Ala) (Figure 4). While the most specific, this is the least druggable site. Although a number of binders to the second and third sites have been identified in silico by our group, their inhibitory effect on Myc-Max transcriptional activity was weaker relative to compounds binding at the Myc-Max/DNA interface (the first site), and as such their functional characterization has not been fully pursued.

Through “blind docking” and clustering analysis, Jiang et al. [150] identified three main sites, coined “site 1, site 2, and site 3” utilizing both the ordered Max-Max [74] and Myc-Max [75] X-ray structures to discover in silico specific Max-Max stabilizers that inhibited Myc-Max function (as described in the Section 3.1.3). “Site 1” was represented by a deep concave protein surface between the two basic helices of the Max-Max dimer at the DNA interface. VS results for the Max homodimer and the Myc-Max heterodimer were similar for site 1, which is consistent with the high degree of structural similarity between the dimers in the basic region. Its high binding strength was attributed to both the strong electropositive potential of several basic residues: Arg35, Arg36, and Arg60 from both Max monomers, and His38 and Lys40 from each Max monomer (see Figure 13 for equivalent residues from Myc). NSC131615, as the representative compound for site 1, counteracted the charges while further engaging in H-bonding with residues in the site. “Site 2” was found as a more hydrophobic pocket neighboring site 1. Compounds targeting this site (i.e., NSC292215) interacted with several positively charged residues: His44, Arg47, and Arg60 from the basic region of one chain of Max, as well as Arg35 from the other chain, and with neutral HLH region residues (i.e., Ile63) nearby. Minor inconsistencies between the Max-Max and Myc-Max dimers were observed in the HLH region of this binding site, such as the substitution of Phe922 in Myc for His44 in Max and a shift of the loop backbone in Myc that is caused by an insertion at residue 933 (see Figure 13 for numbering). “Site3” was a shallow C-shaped cavity at the intersection of HLH and LZ regions. The prominent structural feature for site 3 binding compounds, representative of which is compound NSC13728, was the presence of rigid, flat moieties that fitted within the relatively narrow pocket between LZ residues Tyr70 and Arg75 and HLH loop residue Pro51, with Met74 and Asn78 neighboring the site. Arg75 was the only positively charged residue near the binding site. In the docking predictions, site 3-binding compounds rarely engaged in hydrogen-bonding with side chains neighboring the cavity. A major disparity was observed between site 3 identified on the Myc-Max dimer relative to that of Max-Max. In the latter complex, the pocket used by site 3-binding compounds was blocked in the Myc-Max structure by Max residues Arg254 and Gln261, disfavoring compound binding to this site in Myc-Max. Max-Max stabilizers at site 3 were predicted to dock instead of various minor sites in the Myc-Max HLH region.

The latter aforementioned computational studies provide evidence that ordered dimer structures are more promising in silico targets for therapeutic inhibition over disordered monomeric forms. Computational approaches have the intrinsic power to accelerate the discovery of novel Myc-Max inhibitors by taking advantage of very large chemical databases emerging in recent years, more accurate methods of binding affinity estimation, improved force fields, and state-of-the-art molecular dynamics simulations.

### 3.2. Small-Molecule G-Quadruplex Stabilizers

An alternative and attractive approach actively pursued toward direct Myc therapeutic inhibition is targeting the morphing DNA topologies within the *cis*-regulatory sequences upstream of the *MYC* promoter that regulate *MYC* gene expression. A well characterized nucleic acid regulatory sequence, located -142 to -115 base pairs upstream of *MYC*’s P_1_ promoter, is the NHEIII_1_ element that controls 80 to 90% of Myc expression [164]. Its guanine-rich (G-rich) strand contains a 27 base pair sequence, 5′-TGGGGAGGGTGGGGAGGGTGGGGAAGG-3′, termed Pu27 [165], which is comprised of five consecutive G-tracts that are known to adopt G-quadruplex structures (G4), alternative non double-stranded-B-form DNA (dsDNA) configurations [166]. The typical G4 globular fold consists of three or four stacked guanine tetrads (G-tetrad), three in *MYC* G4 connected by interceding loops formed by up to three adenine or thymine nucleotides. The G-tetrad, the basic unit of a G4, is in turn comprised of four in-plane G bases paired via Hoogsteen-type hydrogen bonding and stabilized by a central monovalent cation, commonly K^+^ or Na^+^, of which potassium is favored due to a stronger coordination at the interface of two G-tetrads [167]. Three different G4 topologies have been observed: parallel, antiparallel, and hybrid or mixed backbone. G4s can arrange in intramolecular (monomeric) or intermolecular (multimeric) structures that are dependent on the number of nucleic acid strands involved in their formation [167]. In physiological relevant solutions containing K^+^ ions, the wild-type Pu27 of *MYC* has been shown to form a dominant intramolecular “propeller-type” parallel-stranded G4 structure arranged from G-tracts 2–5 at the 3′ end of the sequence [166]. The NMR study of Ambrus et al. [168] further demonstrated that the biological relevant conformation of *MYC* G4 consists of a truncated 22 nucleotide sequence with 3 G→T mutations, 5′-TGAGGGTGGGTAGGGTGGGTAA-3′, termed Pu22 (PBD ID: 1XAV). An alternative G4 formed from G-tracts 1–4 at the 5′ end has also been resolved (PDB ID: 2LBY) [169]. *MYC* G4 formation occurs due to negative supercoiling [170], preventing the binding of *trans*-regulatory proteins: the Sp1 transcription factor that binds dsDNA, and hnRNP K and CNBP single-stranded DNA (ssDNA) binding proteins, thus effectively switching off the *MYC* promoter and attenuating *MYC* transcription [165]. The formation and stabilization of *MYC* G4 structures has been shown to be facilitated and modulated by two additional critical regulatory proteins, nucleolin and NM23-H2 [165].

The presence of repressive G4 structures in the *MYC* promoter region provided new opportunities for *MYC* pharmacological inhibition with small molecule ligands that could specifically trigger G4 formation and stabilization, thereby downregulating *MYC* transcription. Nevertheless, the approach faced similar, if not greater, challenges as those encountered with targeting the Myc-Max protein complex and its transcriptional function: the limited availability of crystallographic data for nucleic acid-ligand complexes, and importantly, the selectivity for *MYC* G4 relative to other G4-driven oncogenes leading to off-target side effects. It has been shown that, besides *MYC*, other major cancer driver genes form parallel G-quadruplex structures (G4s), including *Bcl2*, *VEGFA* (vascular endothelial growth factor A), *c-KIT* (KIT proto-oncogene receptor tyrosine kinase), and *HIF1α*, while other G4 types form in the promoter regions of *KRAS*, *RB1*, *hTERT*, and *PDGFA* genes, as well as at telomeric ends (e.g., *h-Telo*) and ribosomal DNA, all of which differ in their folding patterns, number of G-tetrads, loop length, and composition [167,171].

A large number of G4 inducers and stabilizers have been developed in the past fifteen years mainly through chemical design and synthesis evaluated by a variety of biochemical/biophysical and biological assays for determination of their direct binding to G4, stabilization efficiency, key structural ligand-G4 interactions, and effects on gene expression [171,172]. Three major types of G4 ligands have been described based on the structural organization of the aromatic rings of which they are composed: fused heteroaromatic polycyclic systems; macrocycles; and, non-fused or modular aromatic compounds [172]. Three binding modes for G4 ligands have also been described: external stacking attributed to π–π stacking interactions occurring at the external end of G4, the most energetically favored mechanism; intercalation between 2 G-tetrads; and, groove or loop binding [173]. The design of modular G4 ligands was motivated by the need for more “drug-like” and selective compounds targeting the diverse loop and groove regions of G4s and not only the external end G-tetrads of G4s, the preferential binding mode for fused polycyclic and macrocycle ligands, as observed from available X-ray and NMR structural data of G4-ligand complexes [167,171,172].

The diversity of G4 ligands include perylene G4-inducer compounds, such as PIPER and its later synthesized derivatives, synthetic dyes, and natural alkaloids [173]. Stronger binders reported to act as both inducers of G4 formation and importantly as G4 stabilizers include cationic porphyrins, quindoline derivatives, and metal complexes [173]. The representative cationic porphyrin TMPyP4, an end stacker binding the major parallel *MYC* G4, provided early proof of principle that stabilization of G4 structures could silence *MYC* transcription given its effects in Burkitt’s lymphoma Ramos and CA46 cell lines [165]. A significant effect was observed in Ramos cells, which preserve the *MYC* G4 in the NHEIII_1_ sequence after the well-described chromosomal translocation between chromosomes 8 and 14 (which puts *MYC* transcription under the control of an immunoglobulin heavy-chain gene enhancer), but not in CA46 cells in which NHEIII_1_, along with exon 1 (containing *MYC*’s promoter sequence) are deleted during translocation [165]. The observed repression of *MYC* transcription was further substantiated by a reported decrease in the unfolding of *MYC* G4 driven by NM32-H2 at increasing concentrations of TMPyP4 [174,175]. Unfortunately, TMPyP4 has also been shown to stabilize other G-rich sequences, as well as i-motif structures that form on the C-rich strand of the NHEIII_1_ element of *MYC*, and to convert the parallel *MYC* G4 to a mixed parallel/antiparallel type [176]. To address the poor selectivity of TMPyP4, Seenisamy et al. [177] further designed and synthesized Se2SAP, an analog of TMPyP4 with an expanded porphyrin core, which, in comparison to the parental compound, was less toxic and it was able to convert the parallel topology of *MYC* G4 to a single loop hybrid G4. Se2SAP strongly and selectively stabilized the single external lateral loop of the hybrid relative to other G4-forming sequences (e.g., double-loop hybrid induced by TMPyP4 [176]), telomeric G4s, and double- and single stranded DNA, thus demonstrating selectivity for one particular G4 structure.

The quindoline derivative SYUIO-05, a fused heteroaromatic polycyclic and intercalating compound, was shown to preferentially stabilize *MYC* G4 over telomeric G-quadruplexes, as determined by FRET-melting, PCR stop assay, and isothermal titration calorimetry (ITC) [178]. SYUIO-05 significantly arrested cell proliferation of several cancer cell lines and downregulated *MYC* transcription. Moreover, the compound interfered with the binding of NM23-H2 factor to the *MYC* G4 [178]. Several metal complexes have been reported as G4 stabilizers with weak affinity for dsDNA, including platinum II containing compounds [179,180], which are nevertheless prone to additive toxicity and treatment-induced resistance when used as chemotherapeutic agents [181]. Overall, the aforementioned G4 stabilizers have demonstrated efficacy against tumor growth in cancer cells.

CX-3543, also known as quarfloxin, was the first G4-stabilizer that in 2008 had entered phase II clinical trials for low to intermediate neuroendocrine tumors. [182] Its parental compound, the fluoroquinolone QQ58 was originally designed and synthesized by Duan et al. [183] as a derivative started from the norfloxin antibiotic and gyrase agent, via a series yielding a closer intermediate quinobenzoxazine A-62176 compound with antibacterial topoisomerase II inhibitory activity [165]. As QQ58 acted as both a topoisomerase II intercalator and as a G4 interactor, as determined by NMR and molecular simulations, medicinal chemistry efforts that were conducted by Cylene Pharmaceuticals (San Diego, CA, USA) yielded the optimized CX-3543 that demonstrated significant selectivity toward G-quadruplex over duplex DNA with no residual gyrase or topoisomerase II poisonous activity [184]. While quarfloxin was first considered to be a selective binder of *MYC* G4, its mechanism of action was later attributed to its preferential disruption of nucleolin binding to ribosomal G4 in the nucleolus, resulting in the inhibition of Pol I transcription and rRNA biogenesis and induced apoptosis in cancer cells [184]. CX-3543 had been meanwhile withdrawn for clinical studies of neuroendocrine tumors and its development at Cylene had been discontinued [165]. Nonetheless, medicinal chemistry efforts coupled with cell-based and cell-free screens (Pol I transcriptional assay and EMSA among others in the latter category) were further carried out, and, in 2011, Cylene Pharmaceuticals reported the development of CX-5461, a potent and selective inhibitor of Pol I-mediated rRNA synthesis across a panel of 50 cancer cell lines (average IC_50_ of 147 nM), with no effect on DNA replication, Pol II-driven mRNA synthesis, or protein translation [185]. CX-5461 induced autophagy, but not apoptotic cell death in solid tumor cell lines or in normal cells, as determined by immunocytochemistry-based autophagy and senescence detection assays. Moreover, CX-5461 exhibited potent in vivo antitumor activity in murine xenograft models of pancreatic carcinoma and melanoma upon oral administration at a 50 mg/kg dose once daily or every three days without changes in animal body weight or readily apparent toxicity, positioning CX-5461 for investigational clinical trials [185].

In 2017, Xu et al. [186] reported a novel mechanism of action for CX-5461 (and the structurally-related CX-3543) in that CX-5461 induced DNA damage in DNA-repair deficient cell lines. Moreover, in an in vitro FRET melting assay utilizing three different G4 forming DNA fragments (*MYC*, *c-KIT*, and *h-Telo*), CX-5461 showed strong binding and stabilization of G4 relative to dsDNA. Immunofluorescence experiments with a G4 specific antibody further indicated G4 stabilization in the cellular environment upon treatment with CX-5461 at nanomolar concentrations [186]. The CX-5461-induced DNA damage was observed at G4-enriched genomic sequences, the repair of which required breast cancer (*BRCA*) and non-homologous end joining (*NHEJ*) pathways [186]. Importantly, CX-5461 had a profound anticancer activity in *BRCA* deficient and chemotherapy-resistant (i.e., to standards of care taxane and cisplatin) triple negative breast cancer patient-derived xenografts (PDX) tumors [186]. Currently, CX-5461 is in phase I clinical trials for *BRCA1/2* deficient breast tumors [187].

Attempts to discover selective *MYC* G4 stabilizers have been made recently by Felsenstein et al. (2016) [188], who employed small molecule microarrays (SMM) to screen 20,000 drug-like compounds from ChemBridge and ChemDiv repositories. For selectivity purposes, the SMM screening technique was considered to be advantageous due to its fast throughput, and importantly, incorporation early in the design and discovery process of several unrelated oligonucleotide structures in addition to the targeted G4 Pu27-mer of *MYC*. This unbiased SMM screen complemented by a PCR stop assay yielded compound 1 containing a novel G4-binding benzofuran scaffold, which inhibited *MYC* transcription via a G4-dependent mechanism of action. Direct SPR and reversible thermal melt binding assays showed that compound 1 reversibly associated with the *MYC* G4 with a *K*_D_ of 4.5 μM, with no quantifiable or weaker binding to other G4s formed in the promoters of *KRAS*, *Myb*, *VEGF*, *Bcl2*, and *RB1* oncogenes. Compound 1 arrested cell cycle in G1 phase and was selectively cytotoxic to multiple myeloma G4-containing cell lines, including the L363 cell line, but it had no effects on the CA46 Burkitt’s lymphoma cell line, being resistant overall to G4-mediated *MYC* inhibition [188]. Further evaluation of compound 1 selectivity was demonstrated through gene expression analysis and qPCR experiments using L363 cells, which revealed differential G4 *MYC*-driven profiles between compound 1 and quarfloxin on their effects on levels of Myc downstream expression, with substantial reduction of *MYC*-regulated genes over *RB1*, *VEGFA*, *KRAS*, and *HIF1α* ones [188].

In a follow-up study, Calabrese et al. (2018) [189], aiming at understanding the molecular determinants of binding affinity and selectivity for G4 structures, synthesized a focused library of 25 analogs of compound 1, as reported earlier by the group [188]. The substitution of the methyl group on the aryl amine moiety of compound 1 to a para-trifluoromethyl yielded the best derivative, DC-34, which showed enhanced affinity and ability to downregulate *MYC* transcription in multiple myeloma cells in a G4-depedent fashion without affecting the expression of relevant G4-driven oncogenes. DC-34 had a *K*_D_ of 1.4 μM, as determined by SPR, but a *K*_D_ of 9.4 μM in a fluorescent intensity assay (FIA). In FIA, DC-34 preferentially bound *MYC* G4 over other G4 oligos and it did not bind to dsDNA. Importantly, Calabrese et al. [189] resolved the NMR structure of *MYC* G4/DC-34 bound complex (PDB ID: 5W77) demonstrating that extensive and differing bonding interactions or conformational changes within the tail, loop and G-tetrad elements of the quadruplex govern the recognition and selectivity (correlating with biological activity) of DC-34 for *MYC* G4 relative to other G4s, such as those of *KRAS* and *Bcl2*. DC-34 bound independently and distinctly to 5′ and 3′ ends to form a 2:1 ligand-G4 complex in a similar manner to the previously reported NMR structure of a quindoline derivative ligated to *MYC* G4 (PDB ID: 2L7V) [190].

In 2018, Hu et al. [191] reported the discovery of IZCZ-3, a novel and potent “four-leaf clover-like” compound that specifically stabilized the G4 structure of the *MYC* promoter. Its discovery was based on the design, synthesis, and optimization of aryl-substituted imidazole/carbazole conjugates, capitalizing on two already available chemical scaffolds, triaryl-substituted imidazole developed by the same group that stabilized parallel G4s but also telomeric multimeric G4s [192], and carbazole, derivatives, which have been shown to bind strongly to *MYC* G4 [193]. As assessed by fluorescence spectroscopy, IZCZ-3 showed significant selectivity for Pu22, the parallel *MYC* G4, relative to other representative DNA structures, including antiparallel *HRAS* G4, hybrid telomeric htg22 G4, G-triplex, i-motif, and double- and single-stranded DNA [191]. The specifics of IZCZ-3 interactions with *MYC* Pu22 were further investigated using fluorescence titration (*K*_D_ of 0.1 μM), CD melting, and molecular modeling studies substantiating the stabilization of the *MYC* G4 over the parallel promoter G4s of *c-Kit*, *Bcl2*, and *KRAS*. Molecular docking of the optimized IZCZ-3 electronic structure against three template NMR structures of *MYC* Pu22, *c-Kit*, and htg22 showed that IZCZ-3 selectivity for *MYC* Pu22 was due to the lowest binding energy contributed from optimal end-stacking π–π interactions and placement of the central and positively charged IZCZ-3 imidazole ring in the Pu22 cation channel [191]. Evaluation of IZCZ-3 cellular behavior in a panel of assays, including reporter and exon-specific assays, MTT and real-time cellular activity assays, flow cytometry, RT-PCR, and Western blotting, showed that IZCZ-3 induced G0/G1 cell cycle arrest and apoptosis, inhibiting cell growth and *MYC* transcription due to the selective targeting of *MYC* G4. Moreover, in mouse xenograft models, IZCZ-3 effectively suppressed tumor growth of human cervical squamous tumors [191].

Importantly, also in 2018, Stump et al. [194] resolved the X-ray structure of the major Pu22 *MYC* quadruplex at 2.35 Å resolution (PDB ID: 6AU4), providing new avenues for future rational design of specific small molecules targeting *MYC* G4. The X-ray structure was in good overall agreement with the previously reported NMR structure of Pu22 (PDB ID: 1XAV) [168]. Major differences were observed in the conformation of the 5′ and 3′ flanking nucleotides and loop regions, but not in the G4 core. In the 6AU4 X-ray structure, the T1-G2-A3 trinucleotide changed its stacked conformation that was observed in NMR to an extended one, protruding away from the G-tetrad formed by guanines G4, G7, G12, and G17. The triplet T20-A21-A22 at the 3′-end also extended away from the G6, G10, G16, and G19 tetrad. Large deviations were observed in the position of loop nucleotides, in particular that of T7 [194]. Further CD spectroscopy, performed in similar conditions to those employed in the NMR study of the Pu22-quindoline complex (PBD ID: 2L7V) indicated that *MYC* Pu22 maintained the parallel topology in crystallographic conditions, unlike several cases where different G4 conformations have been observed between X-ray and NMR structures [194]. A comparison with the crystal structures of *c-Kit* (PDB ID: 4WO2) and human telomeric (PDB ID: 4FXM) quadruplexes as well as other NMR structures [166] emphasized that the conformations of the 5′ end and loop regions were the main discriminatory features to capitalize on when seeking the selective targeting of particular G-quadruplex structures [194]. Co-crystallization studies of *MYC* Pu22 with novel anthracenyl isoaxole amides (AIMs) G4 ligands developed by the same group are currently ongoing [195,196].

On the computational front, targeting *MYC* G4 with small molecules is currently in its early stages, although advances have been made in the recent years [197]. Through virtual screening and NMR spectroscopy, Ma et al. (2012) [198] identified a modular natural compound carbamide 1 (out of five analogues) as a G4 stabilizer that differed from previously described G4 binders, both in terms of chemical structure and binding mode, as it demonstrated G4 “groove-binding” specificity.

Using a platform that combined a geometric algorithm implemented in DOCK [199] with three-dimensional (3D) pharmacophore screening of 560,000 publically available compounds from ChemDiv and Specs libraries against an NMR derived structure of *MYC* G4 (PDB ID: 2A5R) [200], Kang and Park (2015) [201] identified three potential *MYC* G4 stabilizers. While the compounds were active in Burkitt’s lymphoma Ramos as well as Hela cell lines, they showed minimal thermal G4 stabilization in FRET melting screening assay.

Following an incremental fragment-based docking approach that was implemented in the Surflex-DOCK platform [202], Hou et al. (2015) [203] identified a novel *MYC* G4 stabilizer from a screen of 28,530 compounds from the Chembridge repository against the NMR 1XAV Pu22 structure, rescored to eliminate intercalators and groove binders to dsDNA by docking against 1Z3F and 1K2S structures. The pyrollopyrazine-derived top hit, VS10, bearing a novel scaffold, while less effective that the control quindoline SYUIO-05 in luciferase-based assays using Raji and CA46 cells, it showed selectivity for *MYC* G4 over dsDNA, as determined by SPR and FRET-based competition assays. MD simulations combined with NMR showed that VS10 stabilized *MYC* G4 by stacking over the 5′ terminal G-quartet [203].

Rocca et al. (2016) [204] used a combination of structure-based pharmacophore screening and docking refinement to identify a novel “dual-specificity” G4 stabilizer binding to both *h-Telo* telomeric and *MYC* promoter G4s. Six 3D pharmacophore models with three or four chemical features, including aromatic ring, H-bonding acceptor/donor, hydrophobic, and positive ionizable, were obtained using LigandScout software [205], with further cheminformatics enrichment and validation, from the 3D coordinates of four human telomeric G4 structures differing in their parallel-folding topology (PDB IDs: 3CE5, 2JWQ, 1NZM, and 3CDM) all complexed with known end stacker active ligands of four different chemical scaffolds. Following their established protocol, ~3 million compounds were screened against the six pharmacophore models. Pharmacophore-matching compounds were merged and filtered on the basis of best fitness and satisfaction of Lipinski’s rule of five to yield 1909 compounds subjected to “ensemble docking” simulations against additional G4 telomeric folding topologies: parallel (PDB ID: 1KF1), antiparallel (PDB ID: 143D), and two hybrid types (PDB IDs: 2HY9, and 2JPZ). Further filtering by ADME properties and similarity clustering resulted in a set of 48 purchasable compounds assessed by CD, FRET-melting, and fluorescence intercalator displacement assays for their ability to interact with and stabilize telomeric G4 as well as parallel G4 of *MYC* (PDB IDs: 2A5P and 2A5R). Compound 56, a fused heteroaromatic naphthyridin-containing derivative had a significant stabilizing effect, not only on parallel telomeric G4 but it was also the best binder to the parallel *MYC* G4 [204]. Docking refinements showed that, in the optimized 56-*MYC* G4 conformation, compound 56 stacked at the 5′ end and associated with *MYC* G4 (2A5R) via π-π stacking interactions with G4, G8, and A12 nucleobases, cation–π interactions with G4 and G17 bases and H-bonding with G4 nucleotide [204].

By employing a rational drug design platform combining in silico, biological and biophysical methods, Bhat et al. (2017) [206] identified a novel carbamoylpiperidinium-containing compound, termed TPP, which stabilized *MYC* Pu27 at the known site at its 5′ end guanine stack. The in silico pipeline included virtual screening of 54,645 chemicals from the Maybridge database, followed by Glide docking in all the three modes (HTVS, SP, and XP) using the Pu27 model built from 2A5P NMR structure, at the 5′ end site, followed by all atom MD simulations in explicit solvent. Analyses of docking binding mode and MD trajectories showed TPP-induced stabilization of the 5′ end capping bases G5, A6, A15 via CH–π interactions, further strengthened by H-bonding interactions with 5′ end G4, G11, and A15, and polar interactions with A6, G11, and A15. Subsequent biophysical analysis using CD, isothermal titration calorimetry, and NMR indicated strong, energetically favorable binding of TPP to the parallel Pu27, also providing atomistic details that are consistent with MD results. Biological assays, including MTT, flow cytometry, RT-PCR, and luciferase-based reporter assays showed that TPP selectively induced apoptosis in T47D breast cancer cells with Myc overexpression, with no effect on normal kidney epithelial NKE cells by a mechanism involving the downregulation of Myc expression (at a 35 μM concentration) by arresting the Pu27 G4, thus interfering with *MYC* transcription [206].

For in-depth coverage on the biology and development of G4 stabilizers as therapeutics or probes, the reader is directed to some recent and comprehensive publications [171,172,197]. As an endnote, while the direct targeting of *MYC* transcription at NHEIII_1_ element is gaining tremendous speed, the indirect targeting of NHEIII_1_/NM23-H2 [207] or FUSE/FBP interactions [208], while therapeutically promising, are currently under-explored.

## 4. Discussion and Future Perspectives

The deregulation of Myc has been linked to nearly all human cancers, making this bona fide oncogenic transcription factor a high-value target for therapeutic intervention. Here, we reviewed the knowledge accumulated to date with respect to Myc physiological and pathological role, the complexity of Myc regulation, and the gain-of-function of Myc observed in many cancers. We also summarized the efforts that were undertaken to tackle Myc-driven malignant transformation and overcome cancer addiction to this major oncogene.

The direct approach of targeting Myc with small-molecule inhibitors represents the main focus of this review. Although several groups took on this challenging task, their efforts did not yet yield an effective drug. Of the two direct strategies that aimed at taming Myc in cancer, targeting *MYC* transcription with G-quadruplex stabilizers, although quite complex, is very promising as it already yielded drug candidates that entered clinical trials. “Drug-likeliness” and therapeutic selectivity are nonetheless major concerns for future development efforts. Targeting Myc transcriptional function, on the other hand, is inherently difficult due to the intrinsically disordered nature of the Myc protein, lack of well-defined pockets on its surface, and obligational dimerization with Max. The structural and functional aspects of Myc-Max dimerization as well as those required for specific DNA recognition and transcriptional activation exploited in drug development efforts were emphasized. The prototype inhibitors of Myc-Max function that have been developed over the years demonstrate the suboptimal safety and efficacy profiles. One major pitfall is their general lack of potency. Their chemical structures and physicochemical properties further restrict their clinical utility. Many compounds bear toxic and/or promiscuous moieties, as well as reactive and/or metabolically labile centers. Some are too large and/or are insoluble in water or are too lipophilic, limiting their permeability and their overall oral bioavailability. We calculated the quantitative estimate of drug-likeness (QED) score [209], using the QED webserver [210], for these inhibitors to predict their desirable “chemical beauty”. 10 out of the 22 quantified compounds had a QED score greater or equal to 0.5, like 75% of orally available drugs, while only seven were ranked as having a desirable drug-likeliness profile with scores above 0.61. Although a low QED score might not rule out the potential usefulness of a small molecule as a drug candidate [209], the estimated drug-likeliness profiles of the Myc-Max inhibitors suggest that the large majority, if not all, requires further optimization to become orally available clinically viable drugs.

It is unfortunate that the precise binding mode for most of the published Myc inhibitors, in particular, the most potent ones, is either completely unknown or not yet proven experimentally. Three major sites have been identified on the surface of both the Myc monomer and the Myc-Max heterodimer, as well as the Max-Max homodimer, in their disordered and ordered forms by experimental and/or computational techniques. While the sites on the Myc monomer consist of contiguous stretches of amino acids, the ones on the dimeric structures are formed by individual residues that are contributed by each monomer. Within the three important regions where all the sites in Figure 13 lie, a number of common residues appear to be critical for binding of small-molecule inhibitors and the disruption of protein-DNA and protein–protein interactions. In the basic-H1 region at the DNA interface: Arg914, Leu917, Lys918, Phe921, Phe922, and Arg925 (and equivalent residues from Max: Arg215, Ile218, Lys219, Phe222, and His223). In the H2-LZ region near the dimerization interface: Lys939, Tyr949, Ser952, Val953, and Glu956 (and equivalents from Max: Arg239 and Tyr249). We highlighted the functional importance of these residues in the context of mutagenesis studies and structural evidence from Omomyc. Both experimental and computational studies conveyed the importance of hydrophobic content as essential for binding and inhibition.

Computational studies provided evidence that ordered dimer structures are more promising therapeutic targets over intrinsically disordered monomeric forms. While greatly complementing experimental techniques, such as X-ray crystallography and NMR spectroscopy, computational approaches are best suited to leverage the ceaselessly increasing chemical space soon in the order of billion compounds (e.g., ZINC15 database [211]), advances in protein structure determination, massively-parallel GPU-accelerated computing and state-of-the-art machine learning algorithms for prediction of novel DNA binding sites, protein–protein or protein-DNA interactions, as well as the generation of novel chemicals.

Although important progress has been made in targeting the challenging complexes of intrinsically disordered proteins, with Myc-Max standing as a primary example, the success of future structure-based drug discovery efforts relies on emerging technologies that are capable of solving protein structures complexed with small-molecule inhibitors at atomic resolution. One such promising technique is cryo-electron microscopy (cryo-EM), which may enable the structure determination of protein targets intractable to X-ray analysis as well as helping to identify key features of protein-drug interactions at high-resolution [212]. Armed with such knowledge, a rational approach to Myc-Max inhibition, combining the most advanced and accurate computational techniques for drug discovery, medicinal chemistry, appropriate formulations, suitable delivery systems, and powerful and reproducible preclinical studies, is highly-likely to provide in the near future the much desired drug candidate to be used alone or in combination for treatment of Myc-driven cancers, and to irrevocably change the paradigm that Myc is “undruggable”.
